# Quality and team care response to the pandemic stresses in high performing primary care practices: A qualitative study

**DOI:** 10.1371/journal.pone.0278410

**Published:** 2022-12-01

**Authors:** Milton Eder, Rachel Jacobsen, Kevin A. Peterson, Leif I. Solberg

**Affiliations:** 1 Department of Family Medicine and Community Health, University of Minnesota, Minneapolis, Minnesota, United States of America; 2 HealthPartners Institute, Bloomington, Minnesota, United States of America; McMaster University, CANADA

## Abstract

**Objective:**

To learn how high performing primary care practices organized care for patients with diabetes during the initial months of the COVID-19 pandemic.

**Participants and methods:**

Semi-structured interviews were conducted between August 10 and December 10, 2020 with 16 leaders from 11 practices that had top quartile performance measures for diabetes outcomes pre-COVID. Each clinic had completed a similar interview and a survey about the existence of care management systems associated with quality outcomes before the pandemic. Transcript analysis utilized a theoretical thematic analysis at the semantic level.

**Results:**

The pandemic disrupted the primary care practices’ operations and processes considered important for quality prior to the pandemic, particularly clinic reliance on proactive patient care. Safety concerns resulted from the shift to virtual visits, which produced documentation gaps and led practices to reorder their use of proactive patient care processes. Informal interactions with patients also declined. These practices’ challenges were mitigated by technical, informational and operational help from the larger organizations of which they were a part. Care management processes had to accommodate both in-person and virtual visits.

**Conclusion:**

These high performing practices demonstrated an ability to adapt their use of proactive patient care processes in pursuing quality outcomes for patients with diabetes during the pandemic. Continued clinic transformation and improvements in quality within primary care depend on the ability to restructure the responsibilities of care team members and their interactions with patients.

## Introduction

The highly contagious severe acute respiratory syndrome SARS-CoV-2, the virus which causes COVID-19, was declared a pandemic by the World Health Organization on March 11, 2021. There were no therapies; diagnostic tests were almost non-existent; personal protective equipment was in short supply. Public acknowledgement and exposure to the virus varied greatly as did public engagement with prevention and infection control strategies. In Minnesota, COVID-19 accounted for daily case averages in July 2020 of 596 infections/day and 5.3 deaths/day. By December 2020, average infections had increased five-fold to 3,114/day and deaths ten-fold to 55.8/day. Deaths in Minnesota due to Covid at the end of 2020 totaled 5,382 or slightly less than 1 death for every one thousand Minnesotans [1].

The pandemic strained many aspects of health care delivery, affecting interactions among primary care clinical staff and patients [2, 3]. However, little information exists about the consequences of this strain on the quality of health care for people with chronic diseases like diabetes, particularly from the perspective of practice staff [4]. While the transition from in-person to virtual patient interactions and the many challenges that accompanied this change have been documented [5, 6], how practices adapted their care management processes to ensure care quality has received little attention.

The Understanding Infrastructure Transformation Effects on Diabetes (UNITED) study is a mixed methods study examining how primary care practices organize the delivery of care for patients with diabetes and produce high quality clinical outcomes. The UNITED study quantitatively compared most primary care practices across the state according to standardized quality measures collected yearly for public performance reports [7]. The UNITED study had also planned qualitative assessments of practice performance by conducting three rounds of interviews with practice leaders. Interviews conducted between June 2018 and Feb 2020 provided qualitative data about what they thought contributed to their practice achieving high-quality clinical outcomes for patients with diabetes. Prior to the pandemic, practice leaders described the regular review of reports to identify the gaps and needs of individual patients, followed by patient outreach, pre-visit planning, and opportunistic care during visits. We grouped these care management processes under the label *proactive patient care* [8]. Returning to interview high performing practices between August and December 2020 provided an opportunity to hear from these same practice leaders about whether disruptions created by the pandemic had changed their care management processes. Our objective was to learn what if anything happened to the proactive patient care processes that practice leaders had previously credited with their high-performance ratings on diabetes quality outcomes as a result of the pandemic.

## Methods

An initial quantitative assessment facilitated the identification of the primary care practices who were producing superior clinical outcomes for patients with diabetes. Clinic selection was based on data all practices are required to collect and report as a result of a 2008 act of the Minnesota Legislature. Data submissions and subsequent reports are managed by Minnesota Community Measurement (MNCM), a nonprofit organization that standardizes measures and data collection statewide [9]; MNCM publicly reports on its web site those standardized measures for all primary clinics in the state. The five treatment goals used by MNCM to assess diabetes care are control of blood pressure at less than 140/90 mmHG; A 1c at less than 8%; statin use for lipid control; non-use of tobacco; and aspirin use unless contraindicated [10]. The measurement of quality for patients with diabetes treats these five clinical goals as a composite measure [11]. This approach to measuring quality remained constant throughout the three rounds of UNITED study interviews. Semi-structured interviews were conducted to obtain from the leaders of high performing practices a description of how the pandemic affected care processes and how they responded. Collectively, the interviews inform this qualitative assessment of quality within high performing primary care practices.

### Practice selection

Given the UNITED study’s focus on identifying specific changeable factors and strategies that are most effective in producing high-quality outcome scores, selection was limited to practices that performed in the top quartile statewide on the composite of the five diabetes quality measures for the most recent year with available data (2018). Selection further included only those practices that returned the Physician Practice Connections–Research Survey in both 2017 and 2019; collected as part of the UNITED study’s mixed-method approach, this survey was used to assess a practice’s systematic use of care management processes [7, 8, 12].

### Recruitment

Out of 27 practices, 18 were deemed high performing. Requests for an interview were sent to the individuals within those 18 practices who had completed the 2019 Physician Practice Connections–Research Survey. All communication occurred through email and phone calls (*i*.*e*., invitations, acceptance, and scheduling of interviews). The UNITED Study’s focus was shared during clinic recruitment. A few practice leaders who wanted to involve other staff with management responsibilities in the interview were accommodated. Interviewees were provided the name, title and credentials, of the individual conducting the interview in advance. Of the 18 practices deemed eligible, one had closed, one had not returned the Physician Practice Connections–Research Survey, one declined, and four did not respond. While 10 interviews were conducted, one interview involved staff from two practices, resulting in feedback on 11 high performing practices. Information on the characteristics of the interviews and the individuals involved is provided in Table 1.

**Table 1:** UNITED study data collection and participant characteristics.

Date	Clinic	Duration (minutes)	Transcript # of lines of text	MD in interview	Gender		Repeat interview	Interviewed by
					MD	Staff (team)		
8/10/2020	A	22	363	No		F	Yes	KP, RJ
8/20/2020	B	16	239	Yes	M		Yes	LS, RJ
9/15/2020	C	23	344	Yes	F		Yes	ME, RJ
9/16/2020	D	21	460	No		M, F, F	Yes†	KP, RJ
9/16/2020	E	20	353	No		M, F, F	Yes†	KP, RJ
9/24/2020	F	35	575	No		F, F	Yes	ME, RJ
9/24/2020	G	16	276	Yes	M		Yes	LS, RJ
10/9/2020	H	24	426	No		F	Yes	KP, RJ
10/29/2020	I^Ꙫ^	25	363	No		F, F	Yes^††^	ME, RJ
12/10/2020	J	39	438	No		F	Yes	ME, RJ

F = Female, M = Male

^†^ 2 additional female staff members

^Ꙫ^ 2 practices discussed

^††^ Leaders managed two practices

### Data collection

Three male members of the study team (KP & LS), two primary care physician researchers and one Ph.D. anthropologist/cultural historian (ME) conducted all the interviews for the UNITED Study. A fourth female study team member scheduled and was present for all the interviews (RJ). Interviews were conducted by the research team members using video conference due to university policy limitations on research interactions during the pandemic. Eight of the ten interviews were conducted by the same research team members who had interviewed the practice leaders before the pandemic.

The interviews began with brief and unscripted personal introductions, accompanied by recognition of each clinic’s achievement of high quality patient outcomes for diabetes. The consent process included reading aloud the following explanation of the study purpose:

The overall goal of the UNITED study is to identify the specific changeable factors and strategies that are most effective in producing high scores on MN Community Measurement measures for patients with diabetes. … In our first two rounds of interviews, our team learned that high performing practices like yours used proactive outreach to patients to address their areas of need as their main strategy. Today we’re interested in how your diabetes care has been affected by the COVID pandemic. I would like to start by asking you to identify yourself and your main role/roles at the clinic.

After obtaining oral consent to conduct and permission to audio record a 20 minute interview, leaders were asked to respond to open-ended questions about changes in quality within the clinic and specifically about changes in diabetes care due to COVID-19 (S1 File). This third interview was patterned after the second UNITED study pre-pandemic interview, with the questions focusing on what changes, if any, occurred in the care management processes due to COVID-19. Field notes made at the discretion of each interviewer were not shared. A professional service transcribed the interviews and identifiers were subsequently removed (S1 Appendix).

### Qualitative data analysis

The coauthors met over four months to analyze the transcripts, following the order in which the interviews were conducted. The analysis relied upon a theoretical thematic analysis, focused at the semantic level, which derived themes by privileging the explicit statements and words used by the interviewees over implicit meanings [13]. Additionally, the researchers recognized a shared theoretical perspective in their conceptualization of the practice or clinic as a system and in their assumption that practices with more care management processes would produce higher quality patient outcomes.

Team members individually reviewed each transcript and created memos, summarizing the semantic or surface meanings in how leaders talked about their high performing practices. To avoid interpretive fragmentation, individual reviewers were limited to generating eight memos per interview transcript. The researcher who conducted the interview led the discussion in which all the interview memos and the text segments they represented were compared, contrasted, refined and finalized. In all cases the team worked to achieve consensus. Discussions often considered whether the text described a direct interaction with patients or interactions only among clinic staff. After designating memos (n = 114) for all interview transcripts, the memos were grouped into one of eight categories (Table 2). A record of the memos by transcript (letter) and text segment (line numbers) and their grouping into categories were manually maintained in Word and Excel files. Finally, the categorized memos were further distilled into themes that describe the collective or shared aspects of how these primary care practices and leaders experienced the pandemic and delivered care. While departures from or exceptions to the semantic themes identified were noted in the results reported, the selection criteria obviated considerations of thematic saturation. The University of Minnesota Human Research Protections Program reviewed, approved and monitored this project.

**Table 2:** Eight categories used to group interview memos prior to developing themes.

CARE SYSTEM—17 memos about involvement of the larger health care system
CHANGE—3 memos about decision making
CHANGE, LONG TERM—4 memos on care changes projected to continue
OUTCOMES—9 memos about data availability and quality scores
PATIENT IMPACT—17 memos about patients accessing care and other patient activities
PROCESS—29 memos about staff care management activities and interactions with patients
RELATIONSHIP—17 memos about clinician, care team and patient rapport and visit type
STRUCTURE—18 memos about facilities, staffing, technology; resources to support patient care

## Results

Ten interviews were conducted with 16 key informants representing 11 primary care practices (S1 Appendix). Informant observations and explanations about how the clinic adapted to the pandemic led to the identification of the following five themes.

### 1. The pandemic disrupted established care management processes, especially those proactive approaches that had distinguished these top performing practices from their less well-performing competitors

One informant characterized the pandemic as

an experiment in itself that proved how much proactive outreach matters…. COVID did stop for an extended period of time that proactive outreach, and our measures have shown that. We actually just in the past month have started that proactive outreach again, and you see, if you’re following just sheer data, those measures start to tick back up. So what we talked about a year ago really holds true, and we did a live experiment with it, whether we wanted to or not, and proactive outreach does a lot for our patients

(H:320–333).

Disruptions due to the pandemic did not diminish these clinic leaders’ commitment to quality. The general consensus was that “…had COVID not happened, our quality overall of the things we’re working on would look much different than it does now (F:263–65).” Quality was

still talked about at all of our meetings, you know, that quality measures—all quality measures, not just diabetes but all quality measures really are being talked about and emphasized that it’s important work that we need to do. … I really hope that we are not negatively impacting patient care by the fact or the way that we have to deliver care right now

(A:247–306).

Staff availability largely determined whether proactive patient outreach processes to generate patient care management reminders continued to be supported. One health system that had furloughed staff considered patient outreach important enough to send out messages on behalf of their primary care practices; the informant who shared this observation expressed concern that centralizing outreach at the health system level would diminish the relationship between the clinic staff and patients (H:296–312). As staff returned and outreach restarted, informants reported a need to reassure patients that it was safe to come into the clinic.

### 2. Virtual visits required rapid innovation but produced documentation gaps that disrupted performance management processes

Informants appreciated the speed at which their health care system stood up the technology to support virtual patient visits as they “closed down to routine visits” for at least a few weeks (G:76). Even after re-opening, they tried “to keep patients out of the clinic and at home. And we’re—the change is seeing them either [using] video or some sort of virtual visit with a quick lab appointment if necessary (I:131–34).”

Practice leaders readily acknowledged they had little or no prior experience conducting virtual visits or organizing diabetes care while limiting in-person interactions with patients. Practices variously involved staff in virtual visits. Some involved only the clinician and patient. Some made staff responsible for situating patients in a virtual exam room; some expected staff to perform patient assessments prior to clinician involvement (H: 158). Only one clinic explicitly reported using the structure of in-person visit interactions for virtual visits: Staff “contact the patient first by phone and go through all the questions before I contact the patient (C:121–38).”

Informants reported patient concern about the quality of virtual visits and uncertainty about payment. Some also described older patients as needing more assistance in setting-up a video interface. Informants expressed a strong preference for video over voice only interactions.

I think you get so much more out of a video call than you do just a phone call. And so I really—I kind of push for those video calls when I can and if they do a phone visit with me then ok, the next one I really want to do video and I’ll help walk you through it. So it takes a little more energy on my part but I think they’re getting used to it. Some are doing multiple, have done multiple video calls with me now by this point because it’s, they realize it’s not so hard. And the technology is getting easier too

(C:309–317).

Regardless of whether staff interacted with patients by video or phone, virtual visits produced documentation gaps [4]. “Our [quality] numbers being down are due to not having an office visit in the clinic because we didn’t have a solution for that early on. So, patients need to be seen in order to meet some of the diabetic criteria, right (I:2268–70)?" Suddenly, “it was difficult for our patients to document their A1c levels, to have them come in to get their blood pressures checked, so I think those were probably the two biggest hurdles that we ran into as far as just documenting our care of our diabetic patients (G:77–81).” Combining virtual with lab-only patient visits did not entirely resolve documentation gaps in patient records, which led practices to suspend their use of quality data reports. The lack of reports with up-to-date patient information severed connections between proactive patient care processes and performance monitoring [14].

### 3. Practices re-organized their use of proactive patient care strategies

While acknowledging “there’s only so much we can do over the computer,” clinic leaders learned that “virtual [video] and phone visits are harder to pre-visit plan for (F:223–24).” In response, interviewees reported increasing their focus on the proactive processes of comprehensive and opportunistic care:

…no missed opportunities, having them—if they come to the clinic for an acute visit and we see that their A1C is due, get that bloodwork done. If they’re due for a foot exam, trying to get those things completed at that time. … we were seeing them for acute visits but realizing that some of their routine needs needed to be met as well, and trying to get them back into the clinic might not happen, so getting them taken care of at that time

(D:181–205).

Sustaining quality metrics while incorporating virtual visits produced a rebalancing of proactive patient care processes: Limiting physical access, literally not asking patients to come into the clinic as would have happened before the pandemic, shifted focus to optimally engaging patients in care when they were physically in the clinic. The expectation that care for patients would be organized across virtual and in-person visits meant that staff shifted workflows from proactive outreach to organizing opportunistic and comprehensive care.

As practices began to open up and encourage patients to return, one clinic intentionally began to move away from their established physician-centric care team structure in supporting proactive patient outreach. Rather than assign patient outreach responsibilities to a care team member for each clinician’s patient panel, this practice assigned outreach responsibilities for all clinic patients with diabetes to a single individual. This innovation represents a unique practice approach to the re-organization of its proactive patient care strategies. Research is needed to determine if and perhaps when centralizing proactive patient care processes improves team function, care coordination and patient outcomes.

### 4. Clinic informants said little about how the pandemic affected patients’ everyday lives

Key informants commented on how the quality of diabetes care was not more greatly affected than quality for other chronic conditions. They noted that the pandemic changed patient access to care, and they shared observations about patient diet and exercise

I would say everyone’s eating and exercise behaviors have taken a serious hit. I think we’re doing a lot of encouragement to have people get outside because we think it’s relatively safe to be outside and the weather generally is pretty good. … I think for some reason the exercise seems to be more of a dramatic factor, which I was a little surprised by. But it is, you know, it’s just something that seems to keep coming up. “Oh yeah, like I usually go to the gym and I can’t go to the gym so I haven’t exercised.” I’m like there’s all these other things you can do that don’t include a gym, so yeah

(B:131–46).

The interviewees also described different patient attitudes toward in-person and virtual visits. Practice leaders did not readily differentiate the way in which the pandemic affected patients with diabetes from other patients.

The informant’s role in the practice appeared to influence comments about the social lives of patients. Clinicians drew on their individual patient interactions when commenting on patient experience. Non-clinician leaders referred to patient satisfaction or patient experience reports, while acknowledging they had not kept up with that data. Silence regarding the influence of COVID on patients’ personal lives, despite our asking about that specifically, may reflect the burden clinic personnel were feeling in trying to cope with stresses in new ways.

### 5. Primary care practices benefited greatly from being part of a larger health system

Clinic adjustments and responses to the pandemic were heavily influenced and supported by the larger health systems of which they are a part. Health systems contributed to decisions on furloughs, office closures, virtual visits, and on where and when to see patients with respiratory symptoms. The systems also provided guidance on clinical documentation, specimen collection and personal protective equipment usage.

It’s been pretty phenomenal. … To have a legal department, to have an infectious department and a risk and safety, those committees, those people are sometimes driving us crazy, but they are keeping us as informed and as safe as they can. So, I’m not out here trying to reinvent any wheels, they get to reinvent the wheel

(J:348–54).

Informants characterized health system guidance on workplace safety in positive ways. “There’s been a whole command center that has helped us through making sure we’re doing things safely, consistently, that we are not in positions where we are making up things as we go … much work has gone on in standardizing that work across all of our practices.” (I:307–340) “… you know what is the current PPE to be wearing, what are the current workflows, because everything has to be [*i*.*e*., is] constantly changing.” (J:361–63) The introduction of video visits, the return of patients back to the clinic, these were all decisions made by the organization (H:285–294).

Study informants also described their clinic’s role in their evolving response to the pandemic.

… we also sat down as a primary care leadership team and said—with clinicians, and said, what is important for our nurses to be gathering? Is it the same as an in-person visit? Are there any aspects that you feel like we need to leave out or we need to add? So we made a grid that all clinicians and leadership agreed upon, and then we trained to that

(H:175–80).

Changes were coming, we would say, on a day-to-day basis, but they were coming on an hourly basis. Okay? ‘This is how we’re going to treat this.’ We’d take our guidance from [the health system]. And then, we’d kind of see how we adapt that to each of our practices, because a larger clinic in [the city] is going to be different than a small rural facility in [Lake Town] or [Town Lake]. So, we’re following their guidance but we’re really seeing how we can make that work

(D:259–66).

While health systems provided essential guidance on safety and care management, including when and how to conduct patient visits, each clinic had to operationalize that guidance. Leaders indicated that guidance on in-person access alleviated some of the challenges to managing patients with diabetes, as the guidance facilitated quick visits for labs or blood pressure checks. However, leaders also reported in-person visits challenged fearful and vulnerable (*i*.*e*., elderly) patients (C:235–41; D:238–40). Within this context, clinicians might ask patients to share home measurements of A1c or blood pressure. One leader commented on the potential to expand to primary care their endocrinology group’s ability to upload clinical values from patients who were at home (A:230–37). Another leader noted their health system decided to allow documentation of patient reported blood pressures in the medical record (H:191–201).

Practice leaders who had indicated an awareness of quality performance before the pandemic continued their commitment to quality during it. Health system guidance in response to the pandemic, which practices interpreted and operationalized, strove for quality by keeping patients safe (*e*.*g*., isolating symptomatic patients, using quick lab only visits) and by supporting patients in meeting diabetes and other quality metrics.

## Discussion

Informants indicated that primary care practice leaders worked closely with their larger health systems in responding to disruptions due to COVID-19 in operations, staffing and patient care management processes. Those leaders, having previously credited proactive patient care management processes for their above average quality performance, attributed a decline in quality during the pandemic to disruptions in how they previously implemented those same processes. With telehealth technology hurriedly put in place before practices had planned for virtual visits, some care management processes within the care system were adversely affected. Specifically, virtual visits produced medical record documentation gaps, leading practices to suspend their reliance on data reports. With fewer opportunities to put hands on patients, care teams honed comprehensive care processes, focusing on when patients were physically in the clinic. In reordering of care management processes while striving to maintain high quality, the majority of these primary care practices each described how they functioned as a complex adaptive system [15–18].

Practice leaders similarly described a practice system comprised of staff, technology, care management processes, patients, and the practice’s relationship to a larger health system. They demonstrated an ability to obtain information or to learn about the outcomes produced by the interacting components, and to make adjustments in how the components interact in striving to maintain and improve high quality patient outcomes. Specifically, practice leader descriptions focused on how the practice adapted their use of proactive patient care processes in response to changes in both internal and external conditions.

Informant observations about how these high performing practices responded to the pandemic confirms the UNITED study meta-framework’s depiction of transformation within primary care [8, 19, 20]. Stage 1 of the three-stage framework is characterized by a clinician-centric approach in which physicians operate in stand-alone or shared practice space; they may coordinate billing but not care delivery. Responsibility for continuity and quality of care resides solely with the clinician and patient. In stage 2 practices, staff work in parallel care teams that make use of standardized care management processes to support individual clinicians and their patient panels [20–22]. Clinic staff become increasingly responsible for making connections across patient encounters, helped by reports derived from the electronic medical record that identify gaps in care. Stage 3 practices continue to transform care team roles and responsibilities. In addition to working on consistency in their implementation of care management processes, stage 3 care teams employ proactive patient care processes to improve quality by identifying and informing individual patients of the care they need and by subsequently determining whether that care was actually provided to the patient [23].

The initial UNITED study interviews, conducted in 2017, included practices that demonstrated low, medium, and high performance on their Minnesota Community Measurement composite diabetes outcome measures [8]. Analysis of those interviews found that leaders of practices with lower quality scores did not talk about proactive patient care processes. Leaders of middle performing practices talked about proactive care as a future activity or as something they were starting to introduce. They did not include proactive patient care processes among the standardized or systematic care management processes they credited for driving practice quality performance. In both framework stage 1 and stage 2 practices, patients remain responsible for initiating care. In addition to standardizing care management processes, leaders of practices in the initial round of interviews that were quantitatively determined to be high performing described staff use of regular reports with reliable patient data to identify, reach out to and engage patients in managing their diabetes [8].

We caution readers that practices are complex and that distinctions between framework stages are not absolute. Our qualitative study of primary care found practice leaders acknowledging that standardization of care management processes along with consistency in staff roles and responsibilities had enhanced their practice’s ability to collect and use data to improve performance on patient outcomes. Practice leaders also recognized that proactive outreach further improved performance on shared quality measures.

Well beyond stage 1, the high performing study practices participating in the UNITED study used standardized care management strategies characteristic of a stage 2 practice, augmented by proactive patient care processes, which we associate with a stage 3 practice: Outreach and pre-visit planning for (diabetes) chronic disease management as well as comprehensive and opportunistic care for minimizing missed opportunities. The practices’ ability to maximize quality and outcomes by reorganizing proactive patient care processes in response to the pandemic exemplifies a Patient-Centered Medical Home transformational goal of the practice learning how to learn [24–26].

In striving to identify specific changeable factors and strategies that are most effective in producing high quality outcome scores, the UNITED study proposed a mixed methods explanatory sequential design. We began with quantitative methods to assess primary care performance based on consistently reported practice outcomes for patients with diabetes in relation to variables like medical group size, practice context (*i*.*e*., urban/rural) [12], and medical home certification [7, 27]. Through this work the study team identified associations between care management processes and quality. Comparisons of primary care practice patient quality outcomes facilitated ranking of high (top quartile), middle (two quartiles) and low (quartile) performing practices, enabling us to learn from practice leaders about the relationship between care management process implementation (*i*.*e*., performance) and outcomes and to look for assumptions when using a survey-based inventory of care management processes to assess clinical quality outcomes [20]. Through repeated, similarly structured interviews and a thematic approach that honed themes to ultimately rely on what interviewees actually said, we acknowledged assumptions regarding the physician-centric care team, which pervades the primary care transformation literature [28–32]. While the study sample involved only a handful of practices, both MD and staff interview participants in high performing practices advocated for incorporating proactive patient care processes to improve quality and for exploring opportunities to further advance care team integration.

The initial impact of COVID on staffing levels increased the likelihood that patients would interact with staff with whom they were not familiar. The practice that assigned patient outreach to a single individual for all patients may be a harbinger in the transition from a stage 2 physician-centric care team to a stage 3 model of centralizing some proactive patient management processes.

The high performing practices participating in this case study are transforming into stage 3 clinics by demonstrating both a critical awareness of the dynamic relationship between care team structure and function and an ability to make changes in that relationship in their pursuit of quality [25, 33–46]. In response to this study’s specific inquiry into the quality of diabetes care, practice leaders indicated that they were focused on producing high quality outcomes for all their patients. The perspective of these practice leaders suggests that researchers should more closely attend to the connections between care team structure and function in seeking to understand how the practice capacity to adapt and implement care management processes influences quality achievement and cost [47–54]. Additionally, practice leader interview observations suggest that studies of care team structure and function should examine the potential of combining in-person and virtual patient visit types to improve quality [55, 56]. Finally, the urgency with which practices adapted care management processes in response to the pandemic produced unforeseen problems, supporting prior research conclusions about the importance of building sufficient time into projects in order to adequately assess primary care practice transformation [57–59].

### Limitations

This qualitative study was conducted within a state that possesses few small and independent primary care practices and may not adequately assess the capacity of these types of practices to support expanded care teams or to sustain proactive patient care processes. This study is further limited by relying on descriptions of changes to sustain quality clinical outcomes without knowing the actual quality performance outcomes for patients with diabetes during the first year of the pandemic.

## Conclusion

Larger healthcare organizations positively contributed to their primary care practices’ response to the management of patient care during the pandemic. Clinic leaders accommodated temporary staffing reductions and the shift to virtual visits by emphasizing different proactive patient care processes to achieve high-quality clinical outcomes for patients, including those with diabetes. Practice leaders further expected to deliver care through a combination of in-person or virtual patient visits. As practices continue to adapt their care management processes to improve quality and patient outcomes, continued refinement in proactive care processes within primary care practices will need to include assessments of care team structure and function.

## Supporting information

S1 File UNITED study semi-structured interview questions. (PDF)

S1 Appendix Transcribed and de-identified UNITED study round three interviews. (PDF)
