# Supplementary material for: Quality and team care response to the pandemic stresses in high performing primary care practices: A qualitative study
Source: PLoS One. 2022 Dec 1;17(12):e0278410. doi: 10.1371/journal.pone.0278410 (PMC9714700; doi:10.1371/journal.pone.0278410)
Supplement: S1 File — (PDF) [file pone.0278410.s001.pdf]

S1 File. UNITED study semi-structured interview questions

1. How has COVID affected the way your practice provides care for patients with diabetes?
2. What changes have you made in your care for patients with diabetes since COVID began?

   Probing details: proactive outreach, patient relationship/interaction. How were changes in care determined? Who was involved? How were changes implemented?
3. Which of these changes have had the greatest influence on your quality of care for patients with diabetes?

   Probing details: Are the changes that have occurred temporary or permanent?
4. How have your patients with diabetes been affected by COVID?

   Probing details: Has it made them more reluctant to come in? How well have video visits worked with them?
5. What kind of help have you received from your health care organization to respond to the COVID pandemic or that improves your diabetes care?

   Probing details: Details, other support wanted
