# Supplementary material for: Quality and team care response to the pandemic stresses in high performing primary care practices: A qualitative study
Source: PLoS One. 2022 Dec 1;17(12):e0278410. doi: 10.1371/journal.pone.0278410 (PMC9714700; doi:10.1371/journal.pone.0278410)
Supplement: S1 Appendix — (PDF) [file pone.0278410.s002.pdf]

S1 Appendix. Transcribed and de-identified UNITED study round three interviews

Interview A 2

Interview B 10

Interview C 16

Interview D 24

Interview E 35

Interview F 43

Interview G 56

Interview H 63

Interview I 73

Interview J 82

The following substitutions were made to de-identify each transcript:

XXXXX, XXXXA, XXXXB ... substitutes for distinct primary care practices

YYYYY -- substitute for the name of the larger health system/organization

KK: Hello, this is KK. Can you hear me?

Kevin: Hello, yes, KK, Kevin Peterson.

KK: Now all I have to do is try to figure out how to get my audio to – or my video to work.

Kevin: All right, it sounds like we have some really odd stuff going on here.

KK: Let's see here. There I am. There we go. Hi. Okay, I think we're ready now.

Kevin: All right, that sounds great. It's very nice to meet you, KK. I'm Kevin Peterson. I'm a family doctor and one of the professors at the university. I think you spoke with Dr. Solberg last year.

KK: I did.

Kevin: I really appreciate it. We had some really wonderful and interesting information come out from that. And we're just going to try to pursue that a little bit to try to see how things have changed over the last year.

KK: Gee, nothing has happened over this last year, has it? *[Laughs]*

Kevin: *[Laughs]* Boy, I tell you it's hard to keep up sometimes.

KK: It absolutely is, I agree.

Kevin: Rachel, could you introduce yourself?

Rachel: I was on mute for a second. I am Rachel Jacobson. I am the project manager for the United study, so I kind of help organize all of these interviews and the surveys that we've been doing around the state and to help keep the project moving forward. So nice to meet you.

KK: Okay, nice to meet you.

Kevin: All right, great so I'm going to go ahead and ask Rachel to turn on – is it okay if we record this? That way we can transcribe it.

KK: Absolutely.

Kevin: Rachel, can I have you turn that on and if you could let me know. Maybe it'll show up? Okay so I'm Kevin Peterson and I'm – we're conducting interviews with the leaders of the [clinic] Family Medicine Practice on August 10, 2020. So the overall goal of the United Study, which we're working on, is to identify the specific changeable factors and the strategies that are most effective in producing high scores on Minnesota community measures for patients with diabetes. As you answer the questions, please don't use any patient or clinic staff names. I'd like to be able to record the conversation. We will transcribe it and remove all personal identifiers. Do we have your permission to allow the recording?

KK: Yes.

Kevin: Our first two rounds of interviews our team learned that high-performing practices or clinics like yours used proactive outreach to patients to address their areas of need as one of their main strategies. We're interested in how your diabetes care has been affected by the COVID epidemic … pandemic. So our first question is going to be how has COVID affected the way your practice provides care for patients with diabetes?

KK: So we have in all of April, a majority of May we moved all of our face-to-face visits to phone visits and video visits. And we had quite a few staff who were put on furlough. We had some providers who were put on furlough. And so outreach beyond the patients that we already had scheduled for appointments dropped off significantly during, I'd say, April and May. June and July we started ramping back up having patients come into the clinic. And then we've been able to have staff return from furlough, and we didn't have as many providers on furlough. So we are able to do a little bit of outreach now for our diabetes patients.

Kevin: Are you – do you anticipate getting back to what you were?

KK: I do. I believe that we will believe to get back to doing a lot more of our outreach. We used to have every one of our staff members reach out to 10 patients a week and we, like I said, significantly dropped the number of staff that we had working here, and that didn't allow for many people to be –

Kevin: How many staff have you lost?

KK: Did we lose did you say?

Kevin: Well, furlough.

KK: Furlough? Let's see. The first – so we've done three phases of furloughing. The first phase we cut everybody's hours by 25 percent. The second furlough – and we've got about 22 FTEs so we had to a 25 percent reduction on 22 FTEs. And then the second furlough they did 100 percent furlough for a smaller number of staff. And I think at that point we were down … I think 8 FTEs? I can't remember if it was 7 or 8 fulltime FTEs of staff. And then the third phase we actually only – we had to do 100 percent furlough again but we had a lower number of staff that we had to furlough. And I think the total for that one was 4.8 or 4.9 FTEs. So that significantly reduced the amount of outreach that we could do.

Kevin: The next question here I have is similar. What changes have you made in your care for patients with diabetes since COVID began?

KK: So the early days of COVID what we did, like I said, is we just addressed the diabetic patients that were scheduled for appointments with providers. We didn't do any outreach. June and July we started doing outreach again making sure that we were identifying the patients that absolutely needed their A1c checked and needed those appointments to get in to see for their foot exam or their eye exam and so that was in June and July and so we're ramping back up at this point.

Kevin: Did that have any effect – so that was your proactive. Did that have any effect on your patient relationships or the interaction that you have?

KK: It did. Patients – well, it's kind of a mixed bag. In some respects with some patients, they didn't want to come in because they knew that they were a vulnerable population. And they didn't want to come in so they were happy about having the video visits. And then we had, I'd say, a smaller percentage in the early days that were really unhappy. They really wanted to have that face-to-face communication and conversation with their providers. Now that we're back in having more providers in clinic seeing patients face-to-face they feel like they're getting better care, and they are happier now that they're able to come back in to see their providers.

Kevin: Okay, the … who was involved in those decisions or how are care changes or changes in care determined or who is involved in that?

KK: It was provider- and patient-driven meaning that providers would go through our quality list. They would look at their quality patient lists to make sure that they were reaching out or had contact with the patients who were at higher risk. And then patients also from their perspective, if they knew that they needed to be seen or if they were having any difficulties, then they would reach out.

Kevin: All right. Were there any challenges or maybe it seems straightforward but how did you implement those changes?

KK: It was difficult to implement the changes and, like I said, staff – or not staff, sorry. Patients are frustrated right now that we have limited access to providers. They understand it and yet they're frustrated at the same time. Senior leaders are the ones that are making the decisions, senior leaders for the practice, the primary care practice. They're making the decisions about how many providers we can have in clinic safety staying socially distanced for staff and for providers. We also have to maintain social distancing in our lobbies to make sure that we’re not going over those six-feet limits. So it really has been senior leadership involvement, you know, the leadership in the clinic involvement and the providers on that end as well as we’re talking about all these changes that need to occur.

Kevin: Just to clarify, when you say “senior leadership” you mean YYYYY leadership outside the clinic?

KK: YYYYY leadership, yeah, correct. It’s the vice-president and president levels of primary care.

Kevin: Okay, which of the changes have had the greatest influence on your quality of care for patients with diabetes?

KK: Not being able to have the patients come in the clinic and not having the amount of staff we need to do that outreach.

Kevin: Do you think any of these changes will be permanent or you’ll be seeing permanent changes? Are they all temporary?

KK: No, I think these are all temporary. The social distancing is the biggest piece that we have to overcome. Our clinic is really – we have a lot of patients that come here, but our clinic is small. We don’t – we aren’t able to socially distance very well in our clinic. And that’s the biggest piece that we can’t quite figure out how to overcome at this point because we do need to space people out. We are able to – we are having like our care coordination, some of our

ancillary departments not work in clinic and so then we can put staff in those offices or providers in those office so we can get the work done when we're here and maintain that social distance so that's the biggest challenge so that's the biggest challenge. I think once we get all of our staff back and we're hoping that everybody will be back working 100 percent of their authorized hours the first part of October.

Kevin: Okay so how do you think in your opinion how do patients with patients with diabetes have been affected by COVID?

KK: In my opinion I think that they probably have been affected mildly and what I mean by that is they're not getting the same level of care because they're not able to come into the clinic. The providers are still talking to them. Our elderly population that don't have video capability I think that they're just getting telephone visits, which I think is a lower level of care. But when they have the video visit, I think that they're getting a relatively good level of care. They're able to have communication. The providers, you know, having that face-to-face with the provider seems to make a difference. I don't think from an outcome perspective when I keep looking, I look at our quality number for diabetic or A1c and we're doing really pretty well. I'm able to look at them on a week-by-week basis to see if we're capturing the patients that were due or that need to come in, that they actually got all of their diabetes care done when they had an appointment. And we're doing really pretty well with that at this point.

Kevin: You mentioned that your phone visits you didn't think were as effective. Could you just tell me a little bit more about your phone visits versus your video visits and how they compared with face-to-face?

KK: I think the phone visit … if you're not looking face-to-face at someone, it's easy to get distracted, right. You can watch your child playing ball or your dog doing something, right. There's distractions. When you're having a face-to-face conversation like you and I are having now, it's easier to stay on track and to focus on the mission at hand. I think that when a provider's able to look at a patient and see how they're talking and seeing how they're interacting, that's a better way to use their assessment skills than just using it over the telephone. I don't think you can make a whole assessment with the patient just on the phone, my opinion.

Kevin: Are you losing the testing and some of the other capabilities, things that keep you up to date with D5?

KK: So we have endocrinology here as well as our primary care, so our endocrinologist is not losing that testing because they have their patients' ability to upload their diabetes. We don't do that in primary care, and I think that that's something that we probably need to look at so that we can monitor their blood sugars on a weekly or monthly basis. I know endocrinology uses that a lot but primary care doesn't, and I think that's something that would be helpful if we were able to do. So I'm imagining and I haven't listened in on a conversation with our providers, but I'm imagining they're talking about their blood sugar levels and their testing. So I'm imagining and I'm trusting that they're doing that because they would do that if they were here in the clinic.

Kevin: What kind of help have you received from your healthcare organization, from YYYYY to respond to the COVID pandemic or that improves your diabetes care?

KK: It's still talked about at all of our meetings, you know, that quality measures – all quality measures, not just diabetes but all quality measures really are being talked about and emphasized that it's important work that we need to do. We need to make sure that we're giving the best care to our patients. That means paying attention to all of their testing results and so we get that from the top down. It's something that we talk about weekly as a leadership team, leadership on my level, at the clinic administrator level to the vice-president level and at the vice-president level to the senior leadership level. So we do this in our strategy deployment meetings that we have every single week.

And then for the clinic level staff we're talking about it on probably a bimonthly conversation, so part of strategy deployment at YYYYY is talking about these important measures and in the outcome for our patients. And so we talk about it weekly and, like I said, it's a two-way conversation. It's both up and down the organization.

Kevin: Okay. Do you think your patients are – have lost appreciation for the seriousness of their diabetes or they have more of an appreciation? Do you know?

KK: I really don't know. I know that the physicians have not lost the – or the providers have not lost the importance of it. I don't think patients really have. I mean we're getting a lot of patient video visits, phone visits so I don't think that patients have lost that.

Kevin: Okay, that is great. I guess I'm going to go back and I think we have two minutes left. Do we have two minutes left?

KK: Yup, I do.

Kevin: Okay, Rachel?

Rachel: Yup.

Kevin: So anything else that you think is important in your delivery? It sounds like you were particularly hit hard on some of the pieces that you had said were particularly important like proactive outreach. Has that – is that something – is it affecting diabetes more than other diseases?

KK: Think about that … now I really haven't looked at the patient outcomes lately to see if I could tell if there's a big difference. When our patients have appointments with their providers, we are following up with all of those diabetic measures that are important. But I haven't gone to look to see how many patients we haven't reached out and touched. I have to perfectly honest. I have not done that. I think it probably is something that now that you've had this conversation with me, I think that it is something that I probably will go back and do and look to see if we're missing a huge chunk of our population or if we're doing a pretty good job of keeping up with the majority of our patients. I'm going to put that on my list to do to go out and look.

Kevin: I don't mean to give you more work. You're busy enough.

KK: No. *[Laughs]* But that's a really good point, though. I really hope that we are not negatively impacting patient care by the fact or the way that we have to deliver care right now. And that's just one of the things that I think I need to look into to make sure that we are not doing that.

Kevin: That's great so I don't think I have any other questions for you.
KK: Okay.

Kevin: The … one of the questions I got from the Department of Health the other day was "Do you think that there are ways that you – do you think that your patients with diabetes are at greater risk? Are there any things that you do for your diabetes patients that you don't do for the other patients?" They were just thinking of an article that was saying that if you go into a hospital with COVID and you have a high blood sugar, that you may be at greater risk.

KK: Okay.

Kevin: But that hasn't come into your radar at all.

KK: No, no, I'm pleasantly surprised that our clinic actually has had very few positive COVID patients. In fact I only know of one, so seriously only one that has come to my attention so that's surprising. It doesn't mean that there aren't any, but they haven't come to my attention so I'm pretty happy with that from that perspective that we don't have very many that I'm aware of I should say.

Kevin: Do you think that there are any permanent changes that will have resulted from the pandemic?

KK: Yeah, I think that there is. I think that we will be delivering care differently. Not every office visit will need to be an office visit or every visit with a provider will need to be an office visit. I think the long-term change is going to be we can deliver care with video when you don't need hands-on. You know, you don't need to be touched for an examination. I think that that's going to be the long-term change, that we're going to have to deliver care differently so we can see more patients that need to be in clinic and need hands-on and do the virtual for the patients that don't necessarily need to have those hands-on appointments. So I think that that's going to be a long-term change for us.

Kevin: Well, thank you very much, KK. I really appreciate your participation here. It's been making a real difference in some of he work that we've been doing. And I hope that we'll be able to improve and better understand how to deliver better care in primary care.

KK: I agree. Thank you.

Kevin: Thank you.
KK: All right, take care.

Kevin: All right, thanks. Bye.

Rachel: You, too.
KK: Bye-bye.
Rachel: Bye.

[End of Audio]

Interviewer: … good quality outcomes and your clinic and so on are the real leaders in that. You're in the top group and improving each year. We think we learned a lot about that that we'll be getting back to the groups and clinics about. But this year _____ _____ *[audio glitch]* disruptions caused by COVID. We're interested in trying to understand how COVID disruptions in care affected the environment in which you were providing that care. So if that makes sense and you're okay with this, we would like to record the conversation again so we'll get all of your good words on this.

Physician: Sometimes this thing is muting me but, yes, that's fine. You can totally record it.

Interviewer: Okay, great so, Rachel, you want to turn on your recorder then? And I'm just going to repeat that for the record here that this is Leif Solberg is interviewing Dr. XX on Friday, the 21st of August. Dr. XX is from XXXXX Clinic and we're doing this virtually. So let me just start out by helping us to understand your role at the clinic. When we talked last year, you described yourself as being one of two physician leaders of your clinic. Is that still the case or is that different?

Physician: So I mean I think we've – I mean, I'd say even in the last – so, yeah, so there – and I apologize. Maybe I misspoke a little bit, but ultimately right now there are six providers. There are two physician assistants. Myself and Dr. QQ are, I would say, the two kind of more senior physicians that are there. And then we have one full partner, I mean, like a newer partner. He's been there now for a couple years, but he's just starting his practice within the last two years. He's newly from residency and he is a fulltime provider and he has a role. It's just not as strong as a leadership role yet in the clinic. And then we also have another part-time physician by the name of WW and she works approximately two days a week and she sees regular clinic patients.

Interviewer: Yeah, good. Thank you. So just have five questions for you. The first is how has COVID affected the way your practice provides care for patients with diabetes?

Physician: I think it has increased our creativity level. I think in regard because there are patients who are nervous. There are patients who are well controlled and probably don't need to come in all the time. And so we're trying to be thoughtful about bringing people in needlessly, but I would say we've just been very, very creative in regards to how do we conjure up these visits or the information that we have so that we have a really good sense that their blood

sugars are well controlled or not. For example, you don't necessarily need an A1c to know if someone's blood sugars are controlled. I have people who call in their blood sugars to me over the course of the week. I know what their A1c is going to, right. So as long as – so you can gather a fair amount of information from these patients without necessarily having a one-on-one physical visit and you'd still have a very good idea as to whether you're moving in the right direction with their diabetic care.

Interviewer: So am I hearing your say that COVID has not been an major disruption for diabetes care?

Physician: Our diabetic care has actually been really, really stable which is even a surprise to me. I mean I figured, you know, just like most clinics, I think most – but we have not found this to be – our numbers have not dramatically changed in the last six months.

Interviewer: Okay, well, that's wonderful. That really makes you unique. So when we talked last time, you identified a particular proactive kind of outreach to patients. You were having your care manager or others contacting patients to ensure they were doing well and staying on track and what has it done for that? Has COVID interfered with that at all?

Physician: It really hasn't changed a whole lot I think because a lot of, you know, because the in-person visits aren't always there. I think there actually sometimes can be more opportunity to make those phone calls if people aren't physically coming to the clinic. People are able to, you know, I feel like the in-person visits are almost always longer than the phone calls are for whatever reason, but that really has not disrupted that process. Sometimes people just choose to not pick up their phone so you call them for, obviously, feedback and there's nothing you can do to control that. I would say we're pretty pesky people, though. I mean the staff that we have are very persistent, so if you ignore the first two phone calls, you're probably going to get a third, fourth and a fifth.

So in the end, we've still had a lot of success in reaching people and, if anything, we probably are even – I mean since COVID began, we're even doing more of that now because if they don't have a true visit with me, we're still following those blood sugars. And then based on those blood sugars, we're adjusting medication. We are doing care using those phone calls all the time. We don't wait to see, you know, if it's very clear that their diabetes is not well controlled, we are not waiting for them to physically come in to make an adjustment in their medication. We will make very

small adjustments and then get feedback the next week. But we will continue to make those adjustments every week until their blood sugars are well controlled.

Interviewer: Great. Has it affected your relationship with patients?

Physician: No. I mean I think for me specifically it’s a lot easier of a process because I have a very well-established patient panel. So I could see if you’re meeting someone for the first or second time that that might change things a little bit. It has not for me. My patients know what they're going to get from me. They know what they're going to get from the clinic. They know … so I would say overall it has not changed anything dramatically.

Interviewer: What about the new doctor, do you think it has affected the relationship with the patients?

Physician: I’m so sorry. I couldn’t hear that last question.

Interviewer: I’m just asking in the case of the doctor who’s new at the clinic and the one who only works two days a week whether this might be harder for them to have a good relationship.

Physician: I think it would. I think it would make a difference because if you don’t have that relationship there, I think people are … it’s just kind of the nature of it. Yeah, I think it probably would.

Interviewer: Okay but basically I’m hearing you say that your systems and your approach with these patients has really not changed much at all except you’re doing more on the phone than before. Is that a fair summary?

Physician: Yeah, absolutely.

Interviewer: Okay. From talking with your patients with diabetes, do you get any sense of how they have been affected by COVID in their lives and (inaudible, glitch in the audio)?

Physician: I would say everyone’s eating and exercise behaviors have taken a serious hit. I think we’re doing a lot of encouragement to have people get outside because we think it’s relatively safe to be outside and the weather generally is pretty good. It seems like if there are older people and they find a rhythm in their exercise and that rhythm gets thrown off, they don’t immediately just go switch over to a different type of exercise. They're just like, “Well, I can’t exercise. I can’t do it” so then I would say eating behaviors I think

there's just a lot of stress out there. I mean people, a lot of them certainly see a lot of stress and I think generally people's eating behaviors have changed. I think for some reason the exercise seems to be more of a dramatic factor, which I was a little surprised by. But it is, you know, it's just something that seems to keep coming up. "Oh yeah, like I usually go to the gym and I can't go to the gym so I haven't exercised." I'm like there's all these other things you can do that don't include a gym, so yeah.

But I would definitely say those are probably the biggest things. As far as like medications and taking those and getting those, that does not seem to be affected. We haven't run into excuses as far as people not being able to get medication covered. We haven't run into big insurance glitches, which you kind of would expect if people are having issues with employment like that would be maybe an issue. That has not come up a lot. It doesn't mean that it's not affecting other patient populations, but those are the – I mean I guess those are the things that I've noticed.

Interviewer: Okay, have they been more reluctant to come in to the office?

Physician: Say again?

Interviewer: Has the COVID worries made them more reluctant to come to the office?

Physician: Yeah, it has and honestly we try to honor those concerns. So unless it's … and generally, though, we've been very successful in obtaining enough information to get a sense as to where their diabetic control is and if we get a week's worth of blood sugar information. And it's not clear as to what's clear as to what's going on, then we just get another week's worth until things do become clear. So we've been able to honor a lot of those concerns and still give people some amount of the diabetic care that they needed anyway.

Interviewer: What about the video visits? Have you been pleased with how that has worked or are there problems with that?

Physician: No, I think the video visits are a silver lining. I think there's obviously – I use three different systems based on what the patient's able to do. I mean, again, I think flexibility has got to be the name of the game right now. And so we use three different systems so that we can connect to someone's cell phone. We can connect to their computer. We can connect in a couple different ways and that has been helpful. But I would say, all in all, the

video visits are just a really nice thing to use. I think, obviously, it's always helpful to physically examine someone. But at the same time, you can get a lot accomplished with those video visits.

Interviewer: Okay, final question. What kind of help have you received from your larger organization from YYYYY in coping with any of the pressures created by COVID on your care?

Physician: You mean me personally or my patient panel or …

Interviewer: Just your clinic, yeah. Is there information or resources that YYYYY has come up to help you deal with this?

Physician: I mean are we talking about diabetes now or are we talking about COVID?

Interviewer: Yeah, no. Diabetes.

Physician: I mean I don't know. I'm trying to think of anything … I guess all of the resources that are supported by the organization itself have been, I mean, have been really in place from the very, very beginning. I don't know that there's anything special that we've done since the COVID piece. I think because that system worked well before and it works really well now, I don't know that we've made any massive adjustments in regards to how to do it. I think, obviously, it's all hands on deck right now and focusing on how to reorganize patient visits, the computer visits, how to get those organized, how to set up testing and things like that. So, obviously, the emphasis and most of the support and focus from the organization has been on COVID itself. I don't know that there's been a lot of conversation about it partly just also because we just have not seen a dramatic shift in regards to how our care has been diminished in any dramatic way for our diabetic patients.

I think if we were seeing them fall off the – like our diabetic numbers falling off the face of the earth, then I think they would probably be like "Whoa, what are we going to do about this?" But that just hasn't happened so I don't know that we've created any new systems or new resources or new strategies to address diabetes because what we've had in place since last year hasn't changed, and it seems to really function well even in kind of a time of crisis.

Interviewer: Okay, great. Okay, that's all the questions I have. Is there anything else you want to say?
Physician: No, that's helpful. That's great.

Interviewer: Super. Thank you so much. Appreciate it.

Physician: Have a great day.

Interviewer: Bye.

Female: Bye, thank you.

Physician: You’re welcome.

[End of Audio]

Interviewer: Keep a good record of interviews. So we'd like to record it if that's ok with you.

Interviewee: Sure.

Interviewer: Great. So this is the formal part that I have to read from the institutional review board. My name is Mickey Eater. I'm conducting an interview with the leaders of the XXXXX Minnesota Clinic. Today is September 15th. This is part of the United study in which we are trying to identify the specific changeable factors and strategies most effective in producing high scores on Minnesota community measurement measures for patients with diabetes. As you answer the questions, please don't use any patient or clinic staff names. Again I'd like to be able to record the conversation. We'll transcribe it and remove all personal identifiers and we have permission to allow the recording. And you're shaking your head yes which is great.

In the first two rounds of interviews we learned about high performing clinics like yours. In fact we spoke with you last year and learned that they used proactive outreach to patients to address areas of need as the main strategy. Today we're interested in how your diabetes care has been affected by the COVID pandemic. So I'd like to start by asking you to identify yourself and your main role at this and potentially other YYYYY clinics.

Interviewee: I'm S. I'm a medical lead for the XXXXX Clinic and also XXXXX and XXXXX clinic.

Interviewer: And at those two clinics you spend still one day a week and you're three days a week at the Rogers clinic.

Interviewee: Three days a week at XXXXX. XXXXX is currently hibernated so we're not seeing patients there at all. And then I spend time at XXXXX but not clinical time. I'm there just as the leader.

Interviewer: And can you say – are you seeing patients in XXXXX?

Interviewee: Yes.

Interviewer: And is that kind of all full time or limited? Are you combining physical visits and virtual visits or –

Interviewee: Yeah. So I do three days a week, a mix of virtual and in person care. And we have providers here each day of the week in XXXXX

seeing patients from 7:00 AM to 6:00 PM, a mix of virtual and in person.

Interviewer: So how would you say that COVID has affected the way your practice provides care for patients with diabetes?

Interviewee: It's varied a lot since the pandemic really started affecting us back in March. For a time we weren't seeing any patients in the clinic except for very limited things that we were seeing them for. And we were doing most of our care for our diabetic patients through virtual visits whether that was phone or video visits. And then bringing them in for labs as needed. It's slowly become more and more in person now. We still have some patients who are resistant to being in person and I feel that these are, generally unless they're having other concerns generally do well with our virtual care so reviewing their sugars and talking about symptoms and then having their labs done. We can cover most of the things that we need to cover for those visits and keep them up to date.

So that's been the biggest, the biggest struggle that the patient who doesn't want to be seen in person because of concerns of COVID and doesn't want to do a virtual visit because of potential payment issues with insurance or even wanting to come in and get labs because that means coming to the clinic also. So that's been a challenge. So we're still reaching out to our patients requesting them to come in proactively.

Interviewer: And so have you – what changes have you made in your care for patients with diabetes if you've made any since COVID began? We're talking about visits. Are there other aspects that have changed?

Interviewee: I think really just how we're doing the visits would be just that we're doing a lot of those virtually now. As long as they're otherwise pretty stable there's not other concerns, things that we have to see. We're – that's our biggest change I guess is just doing some virtual visits for them.

Interviewer: Would you say in general your calendar is as full even though there are virtual visits or are you seeing less of your total patient population?

Interviewee: Initially we were seeing less patients. Initially I would say for March, April definitely seeing fewer people. But since then our schedules are full.

Interviewer: So you previously described a situation where the staff would prep charts three days, ideally three days in advance and prepare and kind of look globally at all of the issues that a patient had. Is that still the strategy? And have virtual visits changed the way that any of that – changed that process?

Interviewee: No. We still chart for three days out for our patients and look at them globally for all of their issues that we need to address. And we do that even for our virtual visits. So even if they’re just going to be a home visit, we’ve got all that information in the chart so that we can help determine if we need to have other visits or other things or if it’s something we can address that day.

Interviewer: So are interactions with the patients different now in terms of who is in the room or how patients flow through the clinic?

Interviewee: Not, the only difference is I don’t have a scribe anymore. I used to have a scribe with me. So I don’t have that anymore. But otherwise it’s very much the same as it was.

Interviewer: So have the scribes been eliminated not just for the leaders in the clinic but at the moment limiting the number of people?

Interviewee: Correct. Right now we don’t have any scribes at our sites because of just spacing issues.

Interviewer: Is the clinic staff still kind of triaging and asking initial questions and interacting with patients as they had previously?

Interviewee: Yeah. Even on the virtual visits they contact the patient first by phone and go through all the questions before I contact the patient. So yeah, it’s very much the same.

Interviewer: Just to clarify when you said they talk to them on the phone is that to kind of minimize the amount of time they’re spending in the clinic or are you asking questions you may have previously asked people?

Interviewee: Well, that I was speaking about like for my video and phone visits, the staff will call them first. For a while we were trialing calling patients for all visits including the face to face visits in the clinic. We were calling the day before and getting all that information so that it would streamline the work within the clinic, less time for the patient to be on site. But we have just – I mean actually this week stopped doing that just because it was getting to be too much

calling and too many calls back. And we weren't finding it was adding a whole lot. But they still call for the virtual visits.

Interviewer: So just in advance of your getting on the phone or on the video conference whichever.

Interviewee: Correct. Yeah. Just right before that they'll call them.

Interviewer: So which of the changes that have occurred – actually let me just go back for a minute. How would you say the virtual visits have influenced the care that you're able to provide?

Interviewee: I think it's helped us quite a bit during this time. For those patients who are really worried about coming into the clinic it's helped us reach out to them. It's made it easier for patients. I have patients who do their video visits from work or sitting in their car on a break. They don't have to leave and come into the clinic. I think it's actually helped me follow up with patients a little bit easier. And hopefully some of those visits can still continue that way in the future because I think overall patients seem to really like it.

Interviewer: Would you say – would some of the patients that – is it kind of virtual or in person or have you found that there's been some patients for whom there's – you just mentioned to convenience of kind of virtual interactions. Are there some that are kind of combining the two or has it been –

Interviewee: There's some that I've done both with already since this time has occurred if that's what you mean where I've done a video visit and we've taken care of some concerns. And then later they come into the clinic so we can check their feet and recheck their blood pressure and do some other things that they need that I need to have hands on with them. And some have requested can we do every other visit video or every other visit virtual versus always coming into the clinic. So I think some people really like that.

Interviewer: Do you have patients that work with kind of glucose monitors and so on? So are you able to get some of that information remotely?

Interviewee: Yeah. Right now I guess I don't have a lot of way to get downloads into the system. But they'll take pictures and send me pictures of their logs or enter information through MyChart and send it to me that way or just review it with me on the phone right now. Some work with diabetic educators and I think they have more ability to do that than I do. So –

Interviewer: Mm-hmm. So given that things have changed what changes do you think have had the largest influence on the quality of care for your patients with diabetes?

Interviewee: As far as currently, like right now our changes?

Interviewer: Right now meaning the last month or so? I get the feeling like you're contrasting early on, everything was very different and now you've kind of settled into a –

Interviewee: Yeah. Yeah. I mean.

Interviewer: A routine if that's fair.

Interviewee: I forgot to mention too that XXXXX closed for a time. Like we had two months where we weren't even seeing patients here. We were seeing them all in XXXXX. So it feels very like I could see very distinct the first six weeks we were all virtual. Then we were in XXXXX about six, eight weeks. Now we're back here. So I think things that have helped us like I said at our virtual visits, especially when we moved sites for a while, patients that didn't want to drive to XXXXX, we did a lot more virtual. Now that we're back in XXXXX I feel like we're back to a little less virtual and more face to face again because they're comfortable coming here when we need them to.

But I think I'll definitely continue the virtual care in the future. I think patients want it and I think it's convenient and I think for diabetics it's good to get a touch base that way and periodically have them come in face to face. So I think that will continue and that's been helpful in our care. And then all the things we've done before, the chart prepping and the contacting between visits is very helpful too.

Interviewer: Are there negative aspects to the kind of transition that you, to virtual care for some patients or –

Interviewee: I think some patients just don't want to do that. They're not comfortable doing the virtual care and they're not comfortable coming in. So I have a couple of patients that just want refills and don't want to do any of those follow ups at this point. And the longer this pandemic is lasting the less likely I can continue to do that. If it was going to be three months that's one thing. But now we're at six months and we don't have our end in sight right now. So it's more people getting comfortable. And I'm trying to get people in now before if we do hit with people back at school and

more back at work if we see more cases then it's going to get harder and harder to bring people in. So yeah.

Interviewer: So how – what have your patients shared with you about how the pandemic has affected them or have they?

Interviewee: They do. Most will talk about it. It's all over the board as far as the fear level some people have where they don't want to leave their houses at all and some people that think it's a hoax and all the way in between. So it's really variable just like you see probably in your own life. It's real variable on how seriously people take it and what they're willing to do in their life, how social to be and how much to get out of their house.

Interviewer: Have you found patients' like self-management changed?

Interviewee: That's been kind of all over the board too. I think there's some people who have really taken this opportunity that they're at home and they're working from home, that they're going to really focus on getting exercise every day and they're going to work on their diet. And I've had some of my diabetics that have lost a lot of weight over the last six months and they've been doing really well. And then there's the others that are feeling really anxious about the pandemic and maybe they're eating more. Maybe they don't want to go out and walk. So I've kind of seen both ends of the spectrum really with that.

Interviewer: So the last question or second to last question, what kind of help have you received from the healthcare organization to respond to the pandemic?

Interviewee: You mean just from YYYYY in general or –

Interviewer: Yes.

Interviewee: Yeah. I think a lot. We've gotten lots of information through the organization, lots of communication regularly especially at the beginning, daily communication about what patients to see, how to manage patients. What if they come in? All of that information. And then all of the hardware that we needed in order to get set up for our virtual visits, for video visits, all the training for that. So I feel like we've gotten a lot of good assistance with that.

Interviewer: Were there any instructions specific to diabetes or patients with diabetes and diabetes care?

Interviewee: We talked a lot in the leadership about what was appropriate to bring in at different times. There were definite guidelines around what patients should be seen in the clinic and which ones we should be doing as virtual. And at the beginning especially it was try to manage these patients virtual unless they insist on coming in. But trying to keep people out of the clinic initially. And then slowly it’s transitioned as we have more information and as we have better PPE and supplies and having that more ability to bring patients in for routine things too. So there was a lot of instruction around diabetes patients and other chronic diseases too.

Interviewer: And that was primarily helpful would you say?

Interviewee: Yeah. I think it was very helpful to have a service mind eye on it on how we should all be doing that versus all of us trying to figure out what we should be doing on our own. It was nice to have that discussion too.

Interviewer: Is there support from YYYYY that you wanted that wasn’t available?

Interviewee: I don’t think so. I mean I think as far as the virtual visits we were – the technology wasn’t quite there when we pushed it out to everybody to start doing virtual visits, the video visits. That’s no fault of anyone. Just everything happened so fast. And that, I wish we would have had better instruction and technology right away. But I think they did the best they could in the situation.

Interviewer: If you have one or two more minutes. Have your patients been challenged by the technology? Have you found some that for Zoom or have relied primarily on phone?

Interviewee: Yeah. There’s definitely some patients who are very resistant to trying the video visit. And they’re much more comfortable with just a phone call. But I think you get so much more out of a video call than you do just a phone call. And so I really – I kind of push for those video calls when I can and if they do a phone visit with me then ok, the next one I really want to do video and I’ll help walk you through it. So it takes a little more energy on my part but I think they get – they’re getting used to it. Some are doing multiple, have done multiple video calls with me now by this point because it’s, they realize it’s not so hard. And the technology is getting easier too so –

Interviewer: Is there anything I haven’t asked you about in terms of how COVID has affected the way you practice your leadership in the clinic that you would want to talk about?

Interviewee: I don’t think so. I don’t think there’s anything else.

Interviewer: Well, I hope that you and your staff are staying well.

Interviewee: Yeah. So far so good.

Interviewer: Well, that’s good to hear and again thank you for your time for agreeing to see us a second time. And it was not just an idle threat. Rachel is on the phone as well. And I promise we will send you the publication based on the interviews we conducted last year in the very near future.

Interviewee: Great. I would appreciate that. Thank you.

Interviewer: Thank you Dr. XXXXX and I hope the rest of your day goes well.

Thank you. You guys too. Stay safe.

Interviewee:

Thanks. Bye bye.

Interviewer:

Bye bye.

Interviewee:

[End of Audio]

Kevin: I'm Kevin Peterson. I'm one of the professors in family medicine at the University of Minnesota.

A: Perfect. Nice to meet you.

Kevin: And this is Rachel Jacobsen.

Rachel: Yeah. So, I'm the project manager for the United study.

A: Perfect.

Kevin: Hello, J. Nice to see you again.

J: Yes. How are you guys doing?

Kevin: Very good. Very good.

J: So, I have –

Kevin: The world is a changed place from what we were before.

J: Yes. Very, very much so. So, I have X with me as well, our RN care manager in XXXXA and XXXXD which was kind of new to the role when we went through this last time. So, now she's got a couple of years under her belt and she's going to do most of the talking. No, I'm just kidding.

[Laughter]

Kevin: He checked with you about that, did he?

J: Yes. Yes.

Kevin: The – that's just fine. And we – none of us have our masks on because frankly I'm all alone and I – and except for you guys I guess we all are.

J: Yes. And I just got done eating so I haven't put mine back on, but we're fairly distanced here, A and I, so…

Kevin: So, thanks very much, J. I don't know if you remember, J, but I'm Kevin Peterson. And Rachel Jacobsen. And we were just introducing to A I'm one of the professors at the university. We're doing a large study for the National Institutes of Health that's looking at all the practices in Minnesota and this is kind of a follow-up just to some work that we did. And I think J and A and I

had an interesting conversation before and we hope to kind of adjust that and move forward with it because so much has gone on. So, it's okay. I'm going to – I have to ask this. I have some stuff to get going on here. It says I'm conducting an interview with the leaders of – well, this is both XXXXA and XXXXX right?

J: Yep.

Kevin: So, should we start – we're going to have to separate them a little bit. So, shall we start at XXXXX?

J: Sure. Sure.

Kevin: All right. So, with the leaders of XXXXX Clinic. And today is the 16th of September. So, the overall goal of the United study is to identify the specific changeable factors and the strategies that are most effective at producing high scores on the Minnesota Community Measures for patients with diabetes. As you answer the questions please don't use any patient or clinic staff names. I would like to be able to record the conversation. What we do is we transcribe it and then remove all the personal identifies and that allows us to analyze it. Do I have your permission to allow the recording?

J: Yes.

Kevin: All right. Thank you. In our first two rounds of interviews our team learned that high-performing clinics like yours used proactive outreach to patients to address the areas of need as their main – one of their main strategies. We are interested in learning how your diabetes care has been affected by the Covid pandemic.

J: So –

Kevin: What –

J: Okay. Is that a question yet or –?

Kevin: Not a question yet.

J: Okay.

Kevin: That's just – so, I'd like to start by asking you to identify your main roles at the clinic. I think I remember what they are but let's go ahead and do it anyway.

J: So, I'm the Clinic Director at – and I work for YYYYY but I have – XXXXB, XXXXX, XXXXA, and XXXXD are my clinics. A, who you've met, is our RN care manager for XXXXB and XXXXX. And when we had met previously we had had – C sat in with our meeting and she was the RN care manager at that time. Has since took a different position with a different organization. So, A is relatively new to her role and we actually – how this, how Covid has affected us has been greatly. But we have been more affected by not having that role, that position filled for about a year. So, A was on staff and we knew that we wanted to get her in that position, so we kind of left that position open for a good 10 to 12 months before she was able to fill into that role.

So, our scores – and I'm kind of curious to know where we sit, because I know last time we were pretty good and they were from the year prior, so I think – I'm curious to know where our scores are at now from that year and see what we need to do further to improve those and so forth. But yes, that's where we're at.

As far as the Covid piece of that, that is – that has, oh, affected us with the people following up on their routine care. March, April, May really got slow. We started doing a lot of video and verbal visits just to see those patients, have that contact. It's not quite the same as in-person, listening to their lungs, listening to their heart, but making sure that we're trying to manage their diabetes the best that we could in that way without having them in clinic. And it has since opened up a little bit. I think July and August have picked up. But we're still not at that pre-Covid level of seeing our patients. So…

Kevin: So, we kind of just went through it, and that was my first question: How has Covid affected the way your practice provides care for patients with diabetes?

J: A lot of verbal and video visits. Without having the RN care manager there people weren't really – patients weren't really wanting to come in. Now we have that RN care manager and things have opened up more, so I think it's – that kind of coincided with people's fear not being as great, so we're seeing a few more of those patients and getting back to helping them manage that – their A1Cs and so forth.

Kevin: When did you lose the care manager?

J: Oh… I – June? June of last year.

Kevin: And then, when did you fill the position? Or – did anybody do it in the meantime? Or not?

J: We had nurses that tried to do some of that. But as far as the education piece and having them come in and going through a lot of that stuff we didn't have that. We were lacking that. I – yeah. A started within the last month into this role.

Kevin: So, just last month?

J: Yep.

Kevin: All right. And so, now you have a full-time care manager as of last month.

J: Yep.

Kevin: Okay. We don't have the data from last year yet because it's not been released by Minnesota Community Measures, so I can't actually tell you. And we won't have the data from this year until –

J: And that's the one that I'm curious on, really. I mean, that's –

Kevin: We all are. We all are. Everybody is – we all want to find out what's going on.

J: Yep.

Kevin: So, she started this month. So, what – have you made any changes – or, what changes have you made in your care for patients with diabetes since Covid began?

J: Do you want to – because A was a nurse in the clinic and she's pretty familiar with being in that staff nursing role before she moved into the RN care manager role. So, I think she could speak to that a little bit. I have a few thoughts, but…

Kevin: Okay, but we're kind of focusing now on Mountain Lake.

J: Yep.

A: So, I would say some of the things that we did without our in-care manager and trying to manage those patients during Covid, no missed opportunities, having them – if they come to the clinic for an acute visit and we see that their A1C is due, get that bloodwork done. If they're due for a foot exam, trying to get those things

completed at that time. That's going to be a – that was a huge thing because we knew that patients were having some worries about coming to the clinic. And then, we also had implemented more routine visits in the morning and then sick visits in the afternoon – and so, the education to patients about that, that had helped kind of make patients realize that we were doing everything in our power to protect them. And so, we had more patients willing to come for their diabetic visits. And let's see…

Kevin: So, were you saying that you were not doing the "no missed opportunities" or that you were –?

A: We are. Yep. And we kind of do that with immunizations as well, which can kind of play into all healthcare in general. Their pneumococcal vaccine and shingles vaccine and flu vaccine, those kind of things. But we try to implement that as much as we can. And then, like I said, that was huge for our routine – we were seeing them for acute visits but realizing that some of their routine needs needed to be met as well, and trying to get them back into the clinic might not happen, so getting them taken care of at that time.

Kevin: Got it.

J: So, the piece that I would add to this and how the – Covid affected that is we tried to reassure the patients that they were coming into a safe environment. And our clinic only has one door for patients, so if somebody was coming in – say they may have had positive Covid or were looking to test, we started driving – having those patients drive behind our clinic and we'd do a curbside test. So, we're trying to reassure patients that we're going to keep that potentially Covid patient out of our clinic and still allow for that kind of sick care to go. But we steered those to the afternoon and we did a lot of our well visits, diabetic patients, RN care manager visits in the morning just because we can't separate – our clinic is not real big. So…

Kevin: How did you determine which patients would come in?

J: Symptom-wise. They would report the screening on the – the reception staff screens them, then they'd go to a nurse. And kind of it really depended on what that provider felt was appropriate. We erred on the side of being conservative. If it was a scratchy throat, runny nose, that type of thing, we were most likely looking to test if able versus having them come in. And it – yeah, most of the time it came to the provider making that decision.

Kevin: Okay. So, that was – so, did it affect your proactive outreach? Were you doing proactive outreach or not?

J: I would say yes just because those patients did that apprehension of coming into the clinic. And they still do. I mean, we did a – YYYYY did a pretty deep dive study that patients feel comfortable with their providers but they're still – I think it was 10 to 20 percent that still aren't seeking the care that they need because of the fear of Covid. So, yes, it did affect our –

Kevin: Probably your – maybe your diabetes patients differently than the rest of your patients? Or not?

J: I would say they're both in the same boat.

Kevin: All right. It seems a funny question, but how did you implement those changes?

J: As far as just scheduling-wise and the –?

Kevin: There were a lot of changes going on. How did you –?

J: Yeah.

Kevin: Was it – did you have a strategy? Or did someone just –? I mean, how –?

J: Well, changes came when this all hit. Changes were coming, we would say, on a day-to-day basis, but they were coming on an hourly basis. Okay? "This is how we're going to treat this." We'd take our guidance from YYYYY in XXXXY. And then, we'd kind of see how we adapt that to each of our clinics, because a larger clinic in XXXXY is going to be different than a small rural facility in XXXXX or XXXXA. So, we're following their guidance but we're really seeing how we can make that work. They were able to separate clinics. Their clinics were big enough.
"Here's your potential Covid patients. Here's your well patients." We don't have that. So, we had to change that there and kind of come up with our plan.

Communication went to the front desk, making sure that they're communicating to the nurses and providers. And then, we – there's a ton of e-mails that would go around, but –

Kevin: When you say communication, do you mean from YYYYY or from patients?

J: Yep. Yep.

Kevin: From YYYYY.

J: Yep. Yep.

Kevin: So, YYYYY would contact the front desk and then they would –

J: It would come through me and – that communication piece would come to me and then I'm going to the front desk and to the nurses.

Kevin: Got it. So, you just did it yourself.

J: It got to the point where – what's that?

Kevin: You did it yourself.

J: Yes. So, there were so much e-mails that tends – people didn't really follow through their e-mails. We got to the point with our providers that I would print out these e-mails and meet with them kind of as much individually as I could just to describe some of the changes that were happening. And I know that the nursing supervisors had gone to the nurses with that information as well.

Kevin: All right. Got it. What are the – what changes had the greatest influence on your quality of care for patients with diabetes?

J: I think that – I don't know if it's a positive. I think I see it as a positive, but having that ability to do those verbal and video visits so we're at least having some of that patient contact versus not having that at all. That's probably our – at that time, it was the biggest change that made an impact. Otherwise, we may not have been able to have some of those visits at all. I mean, it – I watched how – they rolled it out really fast. Every provider got an iPad and they could do it whether it was going to a Facetime, whether it was going to be through MyChart, is what we call our electronic. However it was going to be, there was going to be some sort of contact with those patients, which I feel that you have to have that video or verbal visit to have some sort of continuity of care versus not having any visit at all.

So, that ability that they rolled that out, you could see how those started and then they just spiked and there was a lot of those, and

now they've kind of fallen off. They're still there but we're seeing more people in clinic than what we were at that time. So, that would be the biggest change that was able to affect that care.

Kevin: Are the changes that occurred temporary or permanent? What – how is – what's going to happen?

J: I think they are permanent, but maybe not to that degree, which I always try to look forward to knowing how we can do those video or verbal visits in the future, whether Covid is – continues like this, or if we have a bad weather day and I have a provider that can't get to clinic and there's a patient that still wants to be seen. We can always do video and verbal visits from that from their own home. So, that – that's why I think it's going to be around for a while. I don't think it's encouraged to do that all the time, but if somebody needs care and they can get it somehow through tele-med, it's going to give us that access. So, I think it's going to be around for a while.

Kevin: Okay. Next question. How have your patients with diabetes been affected by Covid?

J: A, I'll let you – because you kind of look at the scores a little bit more than I do.

A: So, I would say that Covid has affected our diabetes patients by obviously not having them come into the clinic, not necessarily coming in when they should be coming in. And like I said, implementing that "no missed opportunities" has been a benefit for us in really that it's implemented with the nursing staff. But I would say – I mean, it has overall every – our hypertension patients, our asthma patients, it's affected – Covid has affected everything. And our numbers have decreased as far as our quality numbers go. And then, of course, me coming into this role, we haven't had a care manager, so some of those things it's hard to say is it down because of Covid or is it down because of no RN care manager?

So, I don't know if that answers your question really. Like I said, it's, I mean– we know that Covid has affected everything. But I would say that us really trying to implement the "no missed opportunities" and then doing the tele-med visits and then also doing our more routine visits in the morning and then our acute visits in the afternoon really helped our diabetic patients know that – kind of have that security that we are protecting them as much as we can. We're not having the sick patients in the clinic. The acute

ones that would come to the clinic would be your UTIs, and then if, of course, they needed to have a chest x-ray or listen to their lungs or something like that and we were testing them for Covid, we would bring them back to the room right away and do all of our sanitary purposes that we needed to at that time. But I think just kind of the education for those patients needed to happen, and that happened through nursing and through all the staff.

Kevin: How do you think your diabetes patients have – how have the video visits worked for your diabetes patients? Have they – what do they think of it? Do you know?

A: I don't know as – because the providers usually have kind of all of those. The nurses don't do too much interaction with that visit. I would say that as far as routine visits we don't do them as much. They're more for the acute visits. But if it was something where the patient didn't feel comfortable coming in, the provider would more than likely see them and would be okay with it. So, but like I said, I can't really speak to that as I am not in that visit. But I would think that a lot of the patients would have liked it, especially if they were concerned about coming into the clinic. At least they're still getting their care. They're still getting their med refills, which we would do anyway. The hardest part, I think, would be the lab draw, that A1C to get that routine, whether they're at three months or six months or whatever the case. And I know our lab doesn't draw in the cars. They didn't do a drive-through lab clinic or anything like that. So, they would have to come in for their A1C if they needed that checked. But…

J: We tried to bring that up to try to get that through, but that never went. So…

Kevin: Blood pressure?

J: Lab draws in the car, if somebody had that apprehension to come in.

Kevin: I see.

J: We would try to do that with protimes and so forth, but we got shot down on that.

Kevin: Okay. But you were probably missing blood pressure too, right? Or did you have a way of getting that? No?

J: No.

Kevin: Okay. So, what kind of help have you received from your healthcare organization to respond to the Covid pandemic or that improves your diabetes care?

J: That's a good question. I think we're – they're very supportive in keeping us up to date on everything that's going on. Yes. But as far as – X is going to help with this one. Because I was going to say there's not –

X: So, YYYYY actually – when they did their registries, and the registries tracks all the patients on your diabetic registries, they added a column in there that allows us to pull patients who are falling through the gap, so to speak. So, if patients who are diabetic haven't been seen in the last three months and don't have an appointment upcoming in the next three months to allow us to find those people – they've kind of fallen off our radar – get them back in, get them their labs, get them their follow-up visits, and reassure them that they can come into the clinic. So, that was a huge piece.

Kevin: Okay. Did they set up your telemetry – or your telemedicine? Or not?

J: Yes. Yep.

Kevin: Okay. Are there any other things that you would want them to do?

X: Go get every patient and bring them to the clinic so they do what they're supposed to. *[Laughs]*

Kevin: Okay.

[Laughter]

All right. Well, that's about all that I had to ask for XXXXX. Is that – anything else you want to tell me about that? Anything special going on?

J: No. I think that's – obviously there will be a little bit of a different narrative for XXXXA. But it's a small clinic that's trying to get as many people seen safely as we can, and that's kind of – like I said, I'm curious to know where our measures are at. So –

Kevin: That'll be good.

J: A is a very good, competitive person who wants to see high scores too. So…

Kevin: So, I'm going to stop the interview that was kind of based on Interview D, and let's turn our attention to XXXXA. And I know it may seem kind of repetitive, but you manage a lot of clinics, so I think sometimes every clinic is its own clinic.

J: Yep.

Kevin: I think the last time we were looking at XXXXA and there were a few – there were some concerns about the loss of some providers that –that probably had some impacts on things. So, I think every clinic is a little different. So, let me – I'm going to go ahead and just restart and say that when you look at XXXXA, how did Covid affect the way your practice provided care for patients with diabetes?

J:

So, a lot of the same things. It will be that patients had that apprehension of coming in. Things that we did that are different in XXXA than they were in XXXX is the fact that we were able to have a little bit more of that separation. So, we talked about that clean and sick. We have a building that we utilized which is across the street and we kind of set up for that well type stuff. So, we were able to continue with our RN care managers. There was some lab draws that we were able to do there, and that was – we did it for a short-lived time. We got it up and running. We kind of figured that there's going to be a need here. We kind of pushed that through and I think we did it for about six weeks.

X:

Yes. Yeah. Six sounds right.

J:

About six weeks where we were able to continue to keep some of those patients in-house coming. I didn't have that availability in XXXXB or XXXXX, the two smaller clinics, and we were able to do that in XXXXA and XXXXD, two of the clinics that we have. So, I think that was a big piece of that. We still did the video and the verbal visits, but being able to have those patients come in into a "safe" environment in their mind was a very positive thing.

X:

I'm going to add to that, that with those, with the – when we would schedule the video and the verbal visits, they actually would come over to this well clinic and they would have their blood pressure done, they would do their intake with their nurse. That helped with the blood pressure piece. Otherwise we weren't getting that and those patients were sticking on our diabetes care gaps with blood pressures that maybe weren't satisfactory.

Kevin: Okay. So, were there changes that you made in your care to the patients with Tracy – excuse me, at Tracy – that occurred since Covid started?

J: Could you repeat that?

Kevin: Were there changes at Tracy that you made in your care – were there changes that you made in your care for the XXXXA patients with diabetes since Covid?

J: Yes. With having them come in. Yet we still did, if they weren't, trying to capture as many video and verbal visits. But our numbers – I could tell you our volumes across the board in Tracy as well had decreased during Covid, that March, April, May, pretty significantly.

Other things that I guess I should mention that – biggest changes that we had made – and this would be for both clinics as well – is if we weren't busy, we'd try to consolidate some of those visits and some of those days. So, we actually closed our clinics two afternoons in each of those locations just because we didn't have the volumes and we were trying to not furlough anybody, not send anybody home, and so forth. So, we did kind of adapt our care that way to try to be busier when we were open. So, that did affect patient care in both locations too.

Kevin: So, who was involved in those changes at –?

J: That would be myself. That comes from leadership as far as doing whatever we can to remain viable and see enough patients to keep our doors open. It was kind of a direction that was given –

Kevin: Did you have your providers there too or just nurses?

J: We didn't have – I mean, we closed the clinic doors at noon on a Tuesday and Thursday, or vice-versa – Monday and – whatever days. I'd have to look back. But no, people continued to receive their wage, but yeah, we just closed the clinic.

X: I think the wellness clinic, that was just nurses. And that was driven by one of our doctors.

J: Yeah.

X: That was his push to get patients into – at least to get eyes on them and get numbers and get –

Kevin: So, you worked with a doctor and then he helped set up the nurses? Or – was he seeing patients there or was it mostly the nurses? Just nurses?

J: No, it was – he's our physician in XXXXD that comes to XXXXA one afternoon a week. It was his idea that we do this. And it was also his idea that we needed to set it up fairly quickly. Otherwise, it would by then run its course. Six weeks later we'd have patients coming back in, so we got that implemented pretty quickly, his idea, and we rolled that out to our nursing staff and our scheduling and all that kind of stuff. So, his idea. We're the ones that implemented it.

Kevin: Okay. I'm just curious, was that your property? Or how did you arrange all of that? Did – was – did you own it? Or you just rented it? Or how did you –?

J: Well…

Kevin: I mean, you just set up a new facility?

[Laughter]

J: It's – no. No. No. It is – we have our – well, it's a wellness center that is owned by a private individual, and I don't know if we have space that's rented there or whatever, but we worked with kind of our building manager and our maintenance here, and then they set up some dividers and we got a table and a computer and stuff over there. So…

Kevin: That sounds great. I mean, it's really innovative. That's great.

J: Yep.

Kevin: I'm sorry to get off on that. I just wanted to – which – so, what are the changes that had the greatest influence on the quality of care for patients with diabetes at XXXA?

X: I would say it was for sure having that clinic open because watching my numbers they didn't start really dropping until May or July, which surprised me because by then patients were coming back into the clinic. So, I think having that access and getting those people still to get the checks marks completed on all the items they need to do for diabetes, that was huge. And then, continuing with – like I mentioned, this – in the registries with those patients that are

not there and working the registries. And again, kind of like A said, if we have somebody coming in for something and you can also get their diabetes stuff done at the same time, if possible, you do that because you aren't going to have them come back in in a week. They won't do it. They're nervous already. So, if you can do as much as you can at one time, that's what's best for them.

Kevin: Did telemedicine differ between those two clinics? How was it – how –?

J: No, that was also another – that was another big piece. It was probably – from what I see looking at the numbers it was probably utilized maybe a little bit more in XXXXB and XXXXX than at XXXXA and XXXXD just because we did have those separate clean clinic areas. But we still had a fair amount of those patients that did utilize that. But…

Kevin: So, that – this was about six weeks it lasted, and then you pulled out. Is this something that could come back and go away? Or is it gone now? Or –?

J: I think everything's on the table. If there ever comes a need of that and people have that – and who knows what this winter will entail – we may look at doing that. I know I've had provider meetings from XXXXB and XXXXX on Monday this week and I have one for XXXXA and XXXXD tomorrow. The big concern is how do we manage this? Is it a flu? Is it Covid? Is it strep? Having these individuals in the clinic or not? So, I didn't have those answers and I don't know if anybody has those answers just because of what our testing supplies will be from what our lab –that's changing a little bit too. We just want to try to be as proactive as we can to not have that intermix of patients that may be potential Covid and still trying to see as many well and diabetic and whoever else that needs to be. So, that may come back. We just don't know where it stands a month from now, two months from now. But I think it helps being through the video and the verbal and the clean clinics in the last year that we're going to be able to implement that pretty quick if we decide to do that.

Kevin: Were there any differences in – between the two groups in how the patients with diabetes were affected? Do you think that one group was affected more or less than the other?

J: I don't this so. But I know X may have – because X, I should mention also, in our absence of not having an RN care manager X would go to XXXXB and XXXXX for us on a monthly basis

just to capture some of those patients that really needed to be seen. So, X has a pretty good insight on that too.

X: That's hard because, like A said, there was that absence of a full-time RN care manager over there, and once a month isn't sufficient to do what you need to do for that role. But I can tell you that seeing what happened in XXXXD and XXXXA with the well clinics, I felt like our numbers and our care didn't really fall as much as I would expect other places did because there was still access, there was still outreach to the patients. The care never really stopped. And if there was some hesitance there, there's a lot of reassurance that went along with it, namely by myself and by nurses who were in the registries saying, "It's okay. You can come in. We have this option or this option," where they didn't have that resource at XXXXX to do that.

Kevin: So, other things being equal as far as costs go, do you think that the clean space was a better solution than the telemedicine?

J: That's a really good question. I think it was more effective. But we still – even if we have an RN care manager full time in XXXXB and XXXXX, we don't have the space to keep that. We looked at some different areas, different entrances to XXXXX, and it – we don't have that ability to do that just because it's such a small clinic. I think it's better managed with that separation, but we don't have that resource in XXXXX. Unless we build something new. I don' know.

Kevin: Well, I – people have got to build stuff.

J: Yeah.

Kevin: I mean, that's one of the things that we'll look for as people start building stuff. They'll be looking for advice like that. Is there any kind of – what kind of help have you received from your healthcare organization for XXXXB to respond to the Covid pandemic or that improves your diabetes care?

J: I think that guidance coming through, whether the changes – initially when everything was being rolled out, I think that was very big. The communication piece is big. I would go back, that was probably more in XXXXA than anywhere that I would have to print out those e-mails and go and speak with those providers, that "This is how this is going to be managed; this is what we're looking for." That was probably XXXXA more than the other places.

X: The column, like I had mentioned for XXXXX, having that column of patients that are missing their diabetes follow-ups or not scheduling and that are kind of falling through the gaps, that was also from YYYYY. That was very helpful. And then, I should add – for both places; both XXXXX and XXXXA both got this, but we kind of in the midst of Covid switched from RN care management to the – more looking at a risk score with that so we could pick out patients that were maybe higher on the risk score, more likely to have adverse health outcomes with their diabetes or whatever chronic disease they may have, looking at their risk for hospital admissions, readmissions, ER use. So, with that, when we worked the registries diabetes has the scores on there for each patient on that registry so we can kind of even highlight that even further to pick out the people that are at the biggest risk for health events with their diabetes.

Kevin: Sorry about that. That's what happens with phones in here. All right. I mean, I – you mentioned this other doctor that helped you set up the other site. Was he part of your organization or the YYYYY organization?

J: Yep. Dr. K. He's the clinic director – or the lead MD for XXXXD, which is also – X –

Kevin: So, would you consider his help from YYYYY or from XXXXD, out of your group?

J: No, he's YYYYY. He's a YYYYY physician.

Kevin: Okay. Anything else that you wanted to tell me about XXXXA? I'm out of questions.

X: I don't know if we talked last time about our Hmong interpreter, but we did lose our Hmong interpreter, which is a huge part of our – I mean, it was so beneficial to have that. So, we're kind of at a loss right now without that. And I think – are they still working on finding –?

J: Yeah. And we're in the process of getting something posted because I think it was pretty unique that she was an LPN, so had medical training, and able to do that interpreting and was a very big part of the Hmong community. It's going to be hard to replace her, hard to replace somebody in that community that has their trust and followed through on their appointments and had all that

communicating. So, we're in the process of trying to identify somebody to fill that role.

X: She really filled –

Kevin: So turnover is – sorry.

X: – the cultural gap between the clinic and that community, which is predominantly what the minority population in XXXXA is. And I think without that it's hard to get people to buy into health care. And she did a fantastic job with that. So…

Kevin: So, that's a real impact of turnover. You did have a – lose a couple of doctors too. Did you – were those –

[Crosstalk]

– or not?

J: Well, we're going to have our first full-time YYYYY physician starting in September 28th. So, it's been a long time since we've had that. We've been – we did hire another nurse practitioner, so we have our own – another of our own providers. So, that's one physician assistant, one two-day-a-week nurse practitioner, one full-time, and another two-day-a-week nurse practitioner. So, our provider base is getting more and more solid than the last time that we've talked, so I feel good about that. I really do. And I think it's – XXXXA has already relied heavily on locum since I've been in this role for five years. And I know that if we have our own providers, even though our locum physicians are very good, if we have our own and the community sees that, that they're staying longer, they're going to trust that care a little bit longer than if they make an appointment and that person's not here for three weeks or whatever. So…

Kevin: That's a lot about relationship, isn't it?

J: Yep. Absolutely.

Kevin: Has the relationship changed with our patients at either site because of this whole pandemic or not?

J: I don't think so. You know what? I'm going to go back to that deep dive study that YYYYY did that was 50 pages long and I read through most of it, and a large percentage, very high, high percentage of those patients trust their caregiver and trust YYYYY.

So, I would lump us in with that. Yeah, it was a very enlightening study just to see that. It's just – some people are still apprehensive, but if they're going to trust somebody, they're going to trust our providers. At least, that's what the study showed.

Kevin: That sounds great. I want to thank you so much. I don't want to take any more of your time. I know that you have a thousand things to do and I really appreciate the time to talk with us. We're trying our best to get this information and help tell the rest of the country about how we can cope in Minnesota and how – what it is we can do to kind of mitigate these problems.

J: When you find the solutions just let us know because we'll be listening.

Kevin: We'll have more information when we get those numbers in too.

J: Thank you.

Kevin: Thank you so much for your help.

J: Yes. Thank you.

Kevin: All right.

X: Thank you.

Kevin: All right. Thanks.

A: Thank you.

J: Yep.

Kevin: Bye.

[End of Audio]

Interviewer: Thank you for joining us. Can you see us?

B: I can.

Interviewer: So I want to introduce Rachel Jacobsen, who is the project manager, coordinates everything who probably worked with you to set up this interview.

B: Hi, Rachel.

Rachel: Hi.

Interviewer: We've been working together on various projects for five years now it seems, give or take. B, you might remember I was out in your clinic in January of this year, and we are close to finishing some analysis of the interviews that we did earlier and we promised to share that with you and we haven't forgotten that.

B: Thank you.

Interviewer: So it's just going to be you today from the sound of it and that's great. Again, thank you for your time. I have to read a brief script in order to satisfy our research ethics responsibilities. And if that's okay, I'll just get started then.

B: Sure, go ahead please.

Interviewer: Great. My name is Mickey Eder. I'm conducting an interview with the leaders of the XXXXX clinic today, September 24th. The overall goal of the united study is to identify specific changeable factors and strategies that are most effective in producing high scores on Minnesota community measures for patients with diabetes. As you answer the questions, please don't use any patient or clinic staff names. I'd like to be able to record the conversation. We'll then transcribe it and remove all personal identifiers that may be mentioned. Do we have your permission to record this conversation?

B: Yes, you do.

Interviewer: Thank you. In our first two rounds of interviews, we learned that high-performing clinics like yours use proactive outreach to patients to address their needs as a main strategy. We're interested in how your diabetes care has been affected by the COVID-19 pandemic, and so that's the primary focus of our reason for talking

today. I'd like to start by asking you to, again, identify your main role or roles at the clinic.

B: Okay, sure, and I also have N. She's one of our clinical leads here that just joined me so she's on the call today and then myself, B, the clinic manager.

Interviewer: Welcome, N.

N: Hi.

Interviewer: So could you describe your roles at the clinic? I know you did previously but …

B: Sure so I'm the manager over the business and clinical staff and I co-manage the providers here. Do you want like what are – how our roles are related to quality or just what we do at the clinic?

Interviewer: If you want to describe how your roles are related to quality, that would be great.

B: Okay so mostly I oversee the numbers. We look at them monthly and follow up to make sure that our staff are working the report. N, do you have a summary of what you do as a lead at (crosstalk)?

So I work with one provider, a family practice provider, so I'm
Interviewer:
responsible for all his reports. And then that kind of goes with the same with all our staff. Their regular providers they work with is kind of the reports they're kind of responsible for. Right now with COVID, we had implemented like an outreach person that's trying to help do some of these reports because we are going off a shorter staff with the COVID and they were trying to get these reports done for some of the staff that haven't had the time because they're busy or less staff than we normally had in the past, I guess.

So could you say a little bit more about that one position?

Interviewer:

It's called an outreach position.

N:

So we track the numbers every week. They try and call most of the
B:
patients. They'll send MyChart messages occasionally, too, if they're not able to reach them but anybody that falls onto our reports that needs to be seen. And so we track the number of patients contacted and the number of appointments actually scheduled. Each week we look at that so …

Interviewer: So maybe through the planned questions we'll get into this a little more. Could you both talk about how COVID has affected the way your practice provides care for patients with diabetes?

N: Well, some people aren't wanting to come in necessarily to get their A1c and we can do some stuff over the phone or virtually for diabetics, But at some point we do need an A1c result to keep managing their care and to manage it appropriately. So I mean we're getting to the point where most providers do at least want them to do a lab only if they don't want to be seen in clinic, and we can do a virtual and then do the lab only. So it was kind of set up for a little bit just doing a virtual and then doing labs later. But now more people are coming in for that diabetic visit so we can get everything kind of full care done at the appointment.

Interviewer: So thinking back to when COVID began so back in – I don't know – March, what changes have you made in care for your patients with diabetes?

B: Well, I think what she mentioned where we adjusted kind of the …

N: Just kind of went off their blood sugar results that they could tell us and manage and give them their meds to make sure that they have their medication and kind of postpone the lab for a little bit because people weren't wanting to come in and we were kind of staying away from people coming in at the beginning. So we were kind of mainly going off of how they were feeling and their blood sugar results that the patient conveyed to the provider.

B: And now that we've kind of realized that COVID is our new normal, we're trying to help patients feel comfortable coming in. And I think that message goes hand-in-hand with asking them to come in like it's safe. We clean. We do a bunch of different things that are visible to our patients to know that it's safe to be here, anything from like cleaning the lobby to routing the patients in a one-way fashion through the entrance and the exit, so we can tell patients that over the phone if they're hesitant to come in. We used to have some designations as clinics within YYYYY. There was a COVID, a respiratory clinic and then a non-respiratory clinic and we were non-respiratory through most of that. So I think that helped keep people somewhat comfortable with coming in. But I don't think the care has changed from our providers, from the providers' perspective, just how we're doing it, yeah.

N: Just how we're doing it.

Interviewer: So two questions really. When you said "non-respiratory clinic" could you just explain briefly what that means? And also when you – did you have a point in time when you didn't – when you told people not to come in early on or have you always been trying to see patients?

B: I think initially when they first shut down, we were asking only essential patients to come in, and a well-controlled diabetic patient probably wouldn't have been considered essential. Someone with chest pain and a history of cardiac problems or something like that would be somebody we'd want seen. So we started with back in March and into April like essential only. And then towards the second-to-last week in April is when we got the clinic designation from YYYYY leadership. And respiratory if you had a cough or a fever, you had to go to a respiratory –

N: Shortness of breath.

B: Or shortness of breath, yeah, you had to go to a respiratory site and anything else came to us or one of the other non-respiratory sites. Anybody that wanted COVID testing, anything like that, that all went to respiratory sites so we didn't do any of that here. And then that – we still encouraged patients to come in for needed care. Like we didn't start having physicals and Medicare wellnesses until probably mid-summer, yeah, like July. So once we got through June, then I think people started getting more comfortable. We removed the designations and started requesting people come in for their regular yearly checkups. And I think part of the reason they did that in addition to the fact that we realized that we were able to do a really good job with social distancing and masking and things like that is that they wanted people that needed that care to come in and get it before flu season because flu season is going to create a whole other issue and complexity to try to figure out how to get patients in and keep them safe because the symptoms, obviously, are related. And we expect that people are not going to want to come in for Medicare wellness or regular checkups through the flu season.

The other thing I would say, too, is I know that we had for our diabetic patients but for most of our patients we did give when COVID first started happening like extended refills beyond what we normally would have. So if you normally wanted to see somebody every three months, you'd give them a three-month supply without being seen or maybe another six months or encourage them to be seen virtually, but that kind of thing so that

we could limit the number of people coming in for something that wasn't considered non-essential.

Interviewer: How did your volume change?

B: So I have a graph if you want to see it. *[Laughs]* But mostly it dipped pretty low in March and April and then May we started to rebound and by June we were back at normal. And actually June, July, August we've been above budget so year-to-date we've actually had more visits than we even budgeted for so that's good for our clinic financially. But, yeah, I think the convenience of the virtual visit and the phone visits have been a huge reason why we've been able to continue care that way.

I will say that I've seen a trend in the last month where almost everybody wants to come in in person. There's a lot less virtual. But I expect that once flu season picks up that it'll go back down. And we were lucky to have – we had six providers already live with virtual capabilities in January, so when we had the COVID – when COVID happened, it only took us about two weeks to get everybody else up out of our 14 providers.

Interviewer: Thank you. When we last talked, you spent time and said that pre-visit planning was really important and that you were continuing to monitor it. And how did the shift to virtual visits – how was that organized or were there changes in this pre-visit planning strategy?

B: Initially so in combination with COVID, obviously, our staffing went down. So some of the pre-visit planning just I think by … with less staff and a different workflow kind of fell off. We're getting back into our regular swing of things now.

N: *[Crosstalk]* There's only so much that can be completed with all the, you know, depending …

B: Yeah so I think it's still happening. It's still the same workflow. I think the difference is how we attack it because if you have somebody coming in for a virtual, you need to records to show that they had their colonoscopy or something, it makes it more difficult to get the ROI signed and things like that. And virtual and phone visits are harder to pre-visit plan for which N might be able to speak to a little bit more so.

N: Yeah, I mean there's only so much we can do with a virtual and phone visit. And with the phone visits right now, it's all provider-driven where they call the patient and essentially we don't even

speak to the patient unless the provider needs something to the end, and then we'll follow back up with that patient. And then virtuals, there's only so much we can do over the computer. We can't get a blood pressure. I mean we can do at-home vitals but that they've given us but … so it's minimal I guess as to what we can get completed or done for virtual visits. So the chart prep for a virtual visit isn't as essential as if they're coming into the office, I would say. It's more vital for us doing …

Interviewer: So is it – go ahead, I'm sorry.

B: I was going to say it's just not as comprehensive when they're not coming into the clinic.

Interviewer: So are there other changes that have influenced the quality of your care in the last six months or so?

B: Outside of COVID? *[Laughs]* I don't …

Interviewer: Is there anything outside of it?

B: *[Laughs]* I think that's been the biggest … yeah, it really threw everything into a totally different, yeah, totally different ballgame. But, no, I think that … I was just pulling up our numbers. I think our numbers for diabetic control are still pretty good. I think that a lot of our other colonoscopy screenings, breast cancer screenings, like that kind of stuff – or colonoscopies, not colonoscopy screening. Anyway, those kinds of things are just not as good, the number and the quality, I think because one …

N: They've been put on hold.

B: Yeah, we missed people and, two, it was put on hold. But, yeah, I don't think – I think had COVID not happened, our quality overall of the things we're working on would look much different than it does now. But the things that were in our control I feel like we've done a good job with like HPV vaccine or asthma patients or diabetic patients. Like those types of things we've done a pretty good job of maintaining.

Interviewer: So would it be fair to generalize that disease management has continued but some of the preventive services have been delayed?

B: Yes. Yes, that's a very eloquent way to summarize my rambling. *[Laughs]*

Interviewer: Well, I'm just following you and I appreciate it. You also mentioned when we spoke last January about the importance of regular face-to-face meetings and working in pods. How has that either continued or changed over the last six months? First of all, I guess does the staff work from the clinic or some of them or how have people been organizing their work?

B: So for providers they're mostly in clinic. Some of them have a few virtual days here or there, but most of them are in person. We don't have any clinical staff that work from home or business staff. It's just too vital to really – we need them here. And the spacing in our clinic has been a big issue so we have I think 39 exam rooms. Maybe there's more now. My – I have a grid so I could look at the grid. But we have converted some of our exam rooms into office space. So we're ensuring that everyone has six feet circular around them. So the pods that used to hold 7 or 8 people now hold 3 or maybe 4 with social distancing.

And then a lot of our providers have chosen to kind of work out of an exam room, which is not ergonomically wonderful. But I think that they're seeing patients and then when they do virtual, they'll sit in the exam room and they're working out of the exam room a lot anyway, so it's kind of a natural shift to have them work out of the exam room. It's not something that we want to continue forever but I think … I have to ask N if she thinks that having the provider not in the pod has eliminated some of that natural organic communication or if it's just a different process.

N: It's just a different process, I guess. I mean we're still relatively right next to them almost between a wall so we can still, you know, or we're walking past that exam room to room a patient. So in our team communication is still essentially the same I think for the most part. We just run into the issue with exam rooms now a little bit but ramping back up but …

Interviewer: Are you running reports with the same frequency and sharing the information? I remember you talking last time about weekly monitoring of chart pre-visit planning, you know, chart prep.

B: So in the last probably month, the other clinical lead that is here – she's not here today. But she's been reviewing the pre-visit planning and then I send a message to the people that didn't do it. *[Laughs]* So encouraging them to do it and things like that and what we have found too, even in our last staff meeting we talked about it, that like YYYYY, for example, will send out IFOBT kits to screen for colon cancer if you're due and if the clinical assistants

don't update the health maintenance when they're doing pre-visit planning for the patients, then sometimes a patient that just had a colonoscopy like a year ago will get one of those kits. And then that increases a phone call with them wondering what they should do with it, and the providers kind of get frustrated. So we reinforced with those examples at our last staff meeting about how important chart prep is. So I think that – and we send out our quality numbers monthly to the providers. And I haven't shared them recently with the clinical staff just because there's a lot of red, and I think it'd be pretty disheartening for them. *[Laughs]* Trying to focus on the process rather than the result right now just to give them some time to get back. I think they take a lot of pride in their work and to see our quality numbers has tumbled I think is – except for N. The provider she works with has 100 percent to all his, so she's our quality expert here. *[Laughs]* But I haven't been sharing that recently.

Interviewer: Well, you mentioned you have less staff right now?

B: Yes.

Interviewer: Can you … please, go ahead.

B: Go ahead. *[Laughs]* So we have … currently we have five positions open and then also are running much less than we were before. So like for budgeted for this year if this had been a normal year, we'd have 22.65 FTEs. I'm assume you guys … do you know what an FTE is? Okay, fulltime equivalent so 22.65 is where we would've been right now and with the five open positions we're running more at like a 13.75.

Interviewer: Is that physicians or nursing clinical staff support or both?

B: That's clinical staff support. Providers, we still have 14 providers. It's 11.6 FTE total for them so we're trying to … there's a combination of factors that I think have affected our staffing, some natural turnover, some forced turnover. *[Laughs]* And then just in general the unemployment benefits being supplemented has made working at our pay range less financially beneficial than staying on unemployment if that makes sense. So we've had a little bit more trouble recruiting staff for that reason, I think.

Interviewer: Do you … thank you for that explanation. Do you think that having the clinical staff in the clinic has helped to reassure patients?

B: I think so. I mean I think just having anyone, having the business staff, having the clinical staff here –

N: Right, I think they were just very appreciative that we were open this whole time and that they could still reach out for care and just questions or whatever it might be to help them.

Interviewer: Thank you. Are there other ways that your patients with diabetes may have been affected by COVID? You mentioned some reluctance to come into the office. That seems to have diminished in the last month or two. You mentioned that the video visits have limited you a little bit. Are there ways in which perhaps relationship to provider or other things may have been influenced?

B: I don't know. Do you have … I guess the only thing I'd wonder maybe is like a newly diagnosed diabetic. Before we used to have … they'd get some …

N: Diabetes education.

B: Yeah, they'd get diabetic education, which I think is done virtually now so they still are able to get that. I haven't really talked to any of the providers recently to see how well that's going. I honestly wonder if the access isn't better with them being able to provide virtual care. *[Laughs]* But having that new diagnosis, getting in with the diabetic educators so they know what to do. I feel like the first thing now they ask is, "What am I supposed to eat?" *[Laughs]* They need guidance before they even meet with the educator to know like, okay, what should I eat tonight when I go home now that I know this? So I think that that relationship … I don't know if it was affected or if it got better or if it's similar. Have you had any new diabetes?

N: I haven't had any new ones, no.

B: Not recently? I'd have to investigate a little bit if you wanted an educated answer. *[Laughs]*

Interviewer: Well, it's helpful to just even hear what you think may be happening and concerns. But last question really, again, we've closed the last interview by asking about the support that the healthcare organization has provided you in responding to COVID. And I wonder if you could talk about how the relationship to the larger organization has either helped or hindered your quality and your patient relationships in the last six months or so.

B: You know, I think our lead position here and I talk a lot about how it's hard to know what you would do if you were in that position and having to make those difficult calls. I feel like overall the decisions that they've made regarding our patients have been spot on considering the circumstances. I don't always agree with everything YYYYY does, but when it comes to patient care, I think they always have the patient's interests at the forefront and I think even willing to take that financial hit and go to respiratory and non-respiratory. I think they just did what was necessary and what was appropriate, and I think they knew going into it that it was going to affect us. I would also say we have a really good digital team so, like I said, we were up in two weeks with 14 providers seeing patients virtually, and I know that that kind of change usually in a huge organization like us it's super painstakingly slow and it was more of a "What do you need to take care of your patients? Here, let's get it for you" and it was not like pulling teeth or anything to get that.

I would say staffing-wise like lowering the staffing numbers when we had less patients it makes sense. I think that we'll be extra tight on staffing moving forward, which also makes sense. I mean I don't think that … I feel supported by them when it comes to patient care. I think that's like a no-brainer for me. I feel like they know what they're doing. They're making the best decisions they can in a very difficult circumstance.

Interviewer: N, do you want to add anything?

N: No, I think she pretty much summed it up there.

B: *[Laughs]* You don't have to agree with me.

N: No, I do agree with you, though.

Interviewer: One or two other questions. Did your designation as a non-respiratory clinic mean that you saw patients from other clinics or did you just perhaps end up having some of your patients go elsewhere?

B: So we saw patients from other clinics and some of our patients had to go to other clinics.

N: *[Crosstalk]* Had to go to other clinics.

B: So like our closest one was the XXXXA Clinic in Minneapolis. They were respiratory so our patients that had respiratory

symptoms went there. And then we had a lot of patients from XXXXB is the closest one to us like distance-wise I guess. XXXXA is in our region. XXXXB is the closest distance-wise if that makes sense. I guess you guys may not know the state map *[crosstalk]*

N: *[Crosstalk]* I mean they could also just do a virtual with their primary at whatever clinic they still went to. And then from there if they needed further services like imaging and labs, then they had to go to that designated site, respiratory or non-respiratory so …

B: I think that's why having standard work and pre-visit planning and sharp prep and things like that probably helped take care of the patients, too, because it's an expectation that every pre-visit plans so that the patients that were coming here that weren't our patients got the same treatment that our patients would have.

Interviewer: And we can go back to what you outlined in January about the importance of standardization that would allow you to understand the patient charts and needs of those who perhaps weren't your regular patients.

B: Right, exactly.

Interviewer: One other question. We're probably at 20 minutes and maybe a minute or two over. Has YYYYY or has your healthcare organization – now I'm using names – have they provided assistance for staff in ways that you can outline?

B: Like for staff personally?

Interviewer: Yeah, whether it's emotional, health-related. There's been a big concern about staff mental health and so on during this time.

B: Yeah, we have the Employee Assistance Program, which is like a – I don't know if you know anything about that already, but it offers lots of information on mental health, stress relief, spiritual care, self-care, that kind of thing. YYYYY also has a backup care program, so I've used that throughout this time where they work with QQQQQ and we have additional – normally we get I think 15 days a year to use and I have half a dozen children, so I can send all six of my kids to a daycare for the day and I pay $25.00 and YYYYY pays the rest for 15 days out of the year. And since COVID started, they've offered 20 days instead to help with childcare. They also have free assistance for childcare searches, things like that. They have family resource guides for learning and helping kids stay

active through the summer and through distance learning. What other things?

They have financial … they have a link to help find financial support both with like medical bills and things like that that our patients that are employees can also use. But we also have an employee financial fund, so if out of some awkward circumstance you needed help with your mortgage or your rent or something like that, you can apply for that and I think you can use that twice in your lifetime, maybe once a year and then twice throughout your time with YYYYY.

Yeah, the nice thing about the backup care, too, they also help with adult and elder care. And if your kid is sick, they can send a nurse to your home and YYYYY pays everything except for $6.00 an hour. So I pay $6.00 an hour to have somebody like an RN or an LPN come to my home, and then YYYYY pays the rest so super super awesome. It's been helpful to offer that to people as they're trying to figure out the issues with care with hybrid models for school and distance learning and things like that and there's one pretty close by like two exits away so …

Interviewer: Childcare?

B: Yeah, QQQQQ is just two exits away from us here.

Interviewer: I hadn't heard about those types of programs, so thank you very much. Is there anything you might want to talk about that I haven't asked about in terms of adapting to COVID issues in patient care?

B: No, I don't think so. I think minus the awful negative affect it's having on people's health like COVID for us as a site has really helped us change things in a way that would've taken years to do we've been able to do in a couple months. So not going to make any friends saying that I like COVID, but I like the disruption that it's created because we've been able to adjust how we do our staffing like that outreach position we never would've been able to have if we hadn't had to adjust our staffing. Our hours are slightly different. The providers are very hesitant to make any changes in general have just come along for the ride and really embraced everything. So it's been a change agent here that I think has helped us so I'm a big fan of that piece of it, not …

N: COVID. *[Laughs]*

B: *[Laughs]* Not COVID itself but the disruption that it's caused has been exciting to watch.

Interviewer: We will be sure to maintain that distinction as we're talking about your interview.

B: Thank you. *[Laughs]*

Interviewer: And I thank you both very much and we will send you some of the interpretations as they are finalized from January and subsequently from this interview and just, B and N, thank you both for taking the time and sharing aspects of your current experience in providing care.

B: Absolutely, thank you.

N: Thank you.

Interviewer: Have a wonderful day.

B: You too.

Interviewer: Thanks. Bye-bye. Talk to you later.

B: Bye-bye.

[End of Audio]

Physician: … wearing the mask. I forget I have it on. Yeah, we do all of our –

Rachel: Are you able to see us?

Physician: Yup, can you see me? You probably can't see me.

Rachel: Okay, great. No, we – no, not yet.

Interviewer: You can't see me, Physician?

Physician: Here, I can move this. We do a lot of video. Our system we have here is called video for our video visits so we don't do Zoom. There, you probably – can you see something now? No? Anything now?

Rachel: Not yet. *[Laughs]*

Physician: Not yet. Okay, that's probably … here we go.

Rachel: There we go.

Physician: Okay, good.

Interviewer: Great, good. So, Physician, my name is Leif Solberg and Rachel is the other person on the line here and she set this up. She makes all this work for us. So thank you so much for taking some time. We hope to be done in 20 minutes and let you get a little lunch maybe. Anyway, you may recall having an interview with my colleague Kevin Peterson last November about diabetes care at your clinic. Do you remember that?

Physician: Yes.

Interviewer: So we're back this time with some – a little different slant on trying to understand what's happened with diabetes care in relation to the COVID changes that have disrupted care for a lot of folks, so that's what I'm going to be asking you questions about. And if it's okay with you, we'd like to be able to record like we did last year so we can capture all of your words.

Physician: Sure, that's fine.

Interviewer: Is that okay with you?

Physician: Yes.

Interviewer: Great so, Rachel, you want to turn on the recorder?

Rachel: Yup, all good.

Interviewer: I'm sorry. You got it? It's on. Okay, good so just to start us off here, Physician, could you tell us what your role is at your clinic?

Physician: I'm a family medicine physician.

Interviewer: Do you have any role in leadership there?

Physician: I'm the Medical Director for Population Health at YYYYY and I'm also the Medical Director of the [ ] clinical and training network that we're part of.

Interviewer: Okay, not for your clinic, the XXXXX Clinic?

Physician: Oh, it's overarching so it's all of YYYYY so we're part of YYYYY at our clinic here.

Interviewer: Right, okay, so last time when you talked to Kevin, you identified half a dozen things that you thought were important in the way your clinic cares for people with diabetes. And what I'd like to start out with is kind of getting your overall perspective on how you think COVID has affected the way that your practice provides care for patients with diabetes.

Physician: I think, obviously, there's been challenges with COVID. I think the biggest challenge, of course, is getting people into the clinic. For awhile our clinics and all the rest of them in the state here were pretty much closed down to routine visits. And during that time it was difficult for our patients to document their A1c levels, to have them come in to get their blood pressures checked, so I think those were probably the two biggest hurdles that we ran into as far as just documenting our care of our diabetic patients. I think that through video visits and phone visits, though, we were able to stay in touch with patients, you know, the ones that were monitoring their glucose at home. And I think that was pretty effective as far as helping to titrate meds or insulin with both types of visits even with the pandemic going on so I think there were wins. Some things were wins as far as the being able to do more of the virtual health I think was a big plus that came out of the pandemic here. But I think the big negative was on our quality numbers it was just difficult to document the care that we were providing.

Interviewer: Sure. All right so you already mentioned a little bit here, but what kinds of changes have you made in your care of your patients with diabetes since COVID hit? Have you changed any of the systems or approaches you've used?

Physician: Yeah, I think the biggest one is just more virtual health, more operating the video visits, phone visits. Our diabetic educators also were only working remotely for awhile. Now they're back in the clinics again but, again, I think that in some ways it actually made it easier for patients to get access to diabetic educators that maybe normally would not have done that just because of convenience of being able to do that by phone or video, so I think that's been the biggest change is the telehealth component.

Interviewer: Okay. Last time you talked some about being proactive in reaching out to patients at home about their care and their need for tests and so on. Has COVID affected that? Has it made more or less that outreach?

Physician: Yeah, I think that's a good point too during, especially when things were kind of shut down. We actually had more nursing time to kind of work on what we call our My Panel metrics looking at patients that were in the registry that maybe were deficient in hitting the D5 and reaching out to them, finding out what's going on with them, maybe how we can help them, that type of thing. So some of the My Panel metric, not just for diabetes, we're doing that for other medical conditions as well with their quality metrics looking at My Panel metrics so actually had kind of more time to do that now as we're kind of in this, you know, trying to take care of the backlog plus already being too busy to begin with before COVID hit *[Laughs]* You know, trying to get caught up on everything right now, I think, that there just hasn't been enough time to be going through the My Panel metric like they have previously. So kind of that registry work probably isn't happening as much as it was there for awhile.

Interviewer: Okay, how about the relationship with patients? Has that been harmed or helped by these changes?

Physician: That's a good question. I think it's kind of a mixed bag. I think certainly when we're doing the phone visits and the video visits more, I think patients appreciated being able to connect. I think it helps a lot of patients now that we're doing most of our visits back in person again, I think most patients prefer to have an in-person visit if possible. So I think overall having that availability of the virtual visits was good, but from our perspective, seeing patients in

the clinic I think most of them still prefer to come in for a visit. So I think during that time when they were not able to come for a visit, it was most patients found that not as good as coming in personally.

Interviewer: Yeah, for whatever changes you made in approach to care, how did you do that? Did you have some committee review and plan how you change care?

Physician: Not as far as doing more of the virtual health. That was just kind of every provider, certainly as an organization, we're really pushing providers to really try to adapt as quickly as possible to virtual health. I think we kind of knew it was available, covered by Medicare and a way for us to still connect and provide care to our patients. And so I'm not sure it was necessarily a committee looking at that for diabetes in particular. I think it was just more in general really trying to encourage providers to do as much virtual care as possible.

Interviewer: Okay, which of the changes do you think will show up in having the greatest influence on the quality measures for diabetes care?

Physician: I think where we're probably going to struggle is some patients not having their blood pressure or their A1c documented.

Interviewer: Sure.

Physician: So I think, if anything, it's probably going to be – we're probably going to – if it's going to be a negative, my guess would be in our quality numbers just because of the, again, kind of back to not being able to document the care provided.

Interviewer: Sure. To what extent do you think changes in care approach like virtual care were temporary just during the time of COVID and to what extent will be a new normal?

Physician: Exactly. I think that's kind of where we're trying to find that balance and just not necessarily even diabetic patients but just in general I'd say probably – this is just a guess – but 80 percent of the visits I'm doing right now are in-person and probably 20 percent are virtual. And I'm not sure what that balance is going to be long-term, but I think that's where we're trying to find where kind of that new normal is going to be because there's definitely – virtual care is definitely, hopefully anyway, here to stay because I think there's a huge advantage to it and it has a big role to play in providing care going forward. But at the same time, too, it's not

going to be a total replacement for that in-person care that we provide. So that's kind of a vague answer, but I think we're still struggling with that here on a day-to-day basis trying to figure out where that balance is going to be.

Interviewer: You think it's going to be somewhere in the 20 percent ballpark?

Physician: I think long term it will probably be higher than 20 percent. I think right now it's kind of skewed because we had such a huge backlog – at least in my own personal life here – of physicals that just weren't getting done. And so a big chunk of my day is doing physicals, which we can't, you know, we just can't do virtually. So I think as that backlog probably gets less, then I think long term the percentage of virtual visits is going to be higher.

Interviewer: Okay, switching to your patients, what sense do you have of how their lives and their health and their healthcare has been affected? I'm talking specifically about patients with diabetes now. As you know, they're probably particularly susceptible to COVID.

Physician: Yeah, I think the biggest struggle has just been keeping them connected with the clinic and making sure that they're staying on track with their diabetes care because it's been, you know, with the clinics not seeing patients as much and then now having difficulty with access to even getting an appointment, I think that that's been the biggest struggle is just making sure that patients are staying compliant with their care and monitoring their glucose and staying on their medication plan so I think that's been the biggest struggle. I worry that patients are kind of falling through the cracks and not staying compliant.

Interviewer: Do you think there's still many that are reluctant to come in for visits?

Physician: Oh yeah, definitely and to be honest, probably rightfully so.

Interviewer: Yeah, did you talk to them? Do you get any sense if their lives have been disrupted in big ways like losing their jobs or relationship with family?

Physician: Yeah, as much as, of course, most of my patients are elderly and retired so the job part's not as much of an issue. But as far as the relationship with family, certainly that's always been difficult, especially the patients that are maybe in assisted living or the ones that are a little more frail. They've been really kind of on lockdown right now and so it's been really difficult for them.

Interviewer: Okay and finally you're in a particularly good position to be aware of health from the larger YYYYY organization for what you do in the clinic. Have there been some aspects that YYYYY has been able to provide your clinic support? Have they helped you to cope with these changes with COVID?

Physician: Yeah … I'm sorry. Go ahead.

Interviewer: No, I'm just asking in what ways do you think the larger organization has been helpful?

Physician: Yeah, we've been kind of very thoughtful about adding – that's the other thing I was going to mention, too, one of our physicians up in [city name] that has been working virtually looking through the registries and sending messages to providers on, hey, this patient doesn't have the statin. Maybe think about putting this patient on a statin, you know, no aspirin documented here. Have a conversation about aspirin. So I think that that's been well-received and so he's got a partial FTE to help with going through that registry work with the diabetes and so I think that that's been one area where YYYYY's been quite proactive in trying to say let's look at this registry, have a physician even spend some time going through this and doing some peer-to-peer discussions with providers on trying to improve diabetic control. So I think that's one way where YYYYY's really been proactive on trying to improve diabetic care.

Interviewer: Great. That's pretty much all the questions we have. Is there anything else that you want to tell us about how COVID has disrupted things or what you've done? Have we missed anything?

Physician: No, I think just going through a lot of hand sanitizer every day. *[Laughs]* That the only other big change.

Interviewer: Rachel, you want to add anything?

Rachel: No, I think we covered everything.

Interviewer: Super. Physician, thank you so much for giving us this important 20 minutes and we'll be hopefully able to get back to you and your system with information that will be useful to you.

Physician: Great, thank you. Appreciate your time.

Interviewer: Thank you. Bye.

Physician: Bye now.

[End of Audio]

Interviewee: I'm good. How are you? Good to see you again.

Interviewer: Nice to see you again, too. I really appreciate you being willing to take a few minutes to talk with us again.

Interviewee: Absolutely.

Interviewer: And there's so much going on in the world right now that it's always a little difficult to make sure you can gauge people about this. But because there's so much going on in the world, it's so important to be able to follow what's going on.

Interviewee: Agreed. There's a lot going on, but as time has gone, the change has slowed down, and we're doing okay.

Interviewer: Good. Good. That sounds like – I hear that in other places, too, that it's not like April and May.

Interviewee: That is a good – that's a good benchmark to set perspective nowadays.

Interviewer: Yeah. I'm sure that's true. Well, do you remember RJ? Or maybe RJ wasn't with us last time, were you?

Female: No.

Interviewee: She wasn't. Hi, RJ.

Female: Hi. Nice to meet you.

Interviewee: Nice to meet you.

Interviewer: So RJ works closely – she's a project coordinator and works closely with all of us. And I don't want to – I think we might as well just jump right in.

Interviewee: All right.

Interviewer: So get on with it and get finished up and get everybody back to work and get you back to work.

Interviewee: All right.

Interviewer: So let's – thanks for joining us. So I'm just going to start off, and I need to say that this is KP, and I'm conducting an interview with the leaders of the [Interview H] Clinic, and the

date today is October 9th. The overall goal of the united study is to identify the specific changeable factors and strategies that are most effective in producing high scores on the Minnesota community measures for patients with diabetes. As you saw – as you answer the questions, please don't use any patient or clinic staff names. I'd like to be able to record the conversation, because we transcribe it, and we remove all the personal details, but then we can evaluate it. Do we have your permission to allow the recording?

Interviewee: Yes, you do.

Interviewer: Thanks. In our first two rounds of interviews, we learned that high performing clinics like yours used proactive outreach to patients to address the areas of need as one of their main strategies. We're interested in how your diabetes care has been affected by the COVID pandemic.

Interviewee: So –

Interviewer: Well, let me start. I'll – can I ask – can I start by asking you to identify your main role at the clinic?

Interviewee: Yes. I am the clinic manager, so I oversee all operations in the clinic.

Interviewer: Okay. How has your – how has COVID affected the way your practice provides care for patients with diabetes?

Interviewee: We have ventured into many different ways that we can provide care now. So we are providing some virtual care for our diabetic patients, based on their preferences, so that has definitely changed with COVID. We were not offering that a year ago when we spoke.

Interviewer: Are there – you mentioned some changes – one of the changes – what changes have you made in your care with diabetes since the COVID pandemic started?

Interviewee: We're offering different venues of care. So we're offering virtual visits. We're still, however, staying consistent with the five – Diabetes Five, so keeping up with A1C and blood pressure. But virtual care is an option. Home blood pressures are an option for reporting for our patients. That is a big change, because we used to have patients in the clinic all the time. We took their blood pressures, and we went by that, but now patients can report their blood pressure as well.

Interviewer: Could you describe a little bit more about both your virtual care and your home care?

Interviewee: Yeah. So with virtual care, our patients have two options, video visits or phone visits. It's nice for that connection to still have the video visit, but we realize all of our patients don't have that technology or resources available to them, so we do offer phone visits as well.

And the visit is still focused around diabetes, if that is what is occurring. We still ask the patient to come into the clinic to get an A1C if they are due. And then we also ask for patient reported blood pressures. If they don't have any patient reported blood pressures for a diabetic patient, we would schedule them a nurse visit. So they could come into the clinic, quick in and out, and still get that blood pressure taken, because that is still an important part of our diabetes care, that we have blood pressures, we have the A1C. Obviously, when they're getting the A1C, we would grab a UMAR as well.

Interviewer: I'm sorry, a UMAR?

Interviewee: Yes.

Interviewer: What's that?

Interviewee: Urine –

Interviewer: Microalbumin. Okay.

Interviewee: Thank you.

Interviewer: Right.

Interviewee: So that's a separate measure of Minnesota community measures, but we do catch that – we try to get that yearly with all of our diabetics.

Interviewer: Okay.

Interviewee: So that is mostly how our virtual care is framed up. We do ask our patients with the blood pressures if it's an automatic blood pressure cuff versus a manual. The reality is that a lot of people aren't taking manual blood pressures at home, but we do like to validate that with the patient when we are recording their blood pressures.

So I would say that's kind of a virtual diabetic, in a nutshell.

Interviewer: Who is involved in those visits at your clinic?

Interviewee: Our support nursing staff, which could be an MA or an LPN. They are calling the patient ideally 15 minutes before the provider, and they are doing all of the rooming. So they are collecting those blood pressures, they're talking to the patient about any recent blood sugar readings, and transferring that into the EMR for the provider. And they're doing a lot of that information-gathering before.

And then the provider is also obviously in that interaction, but that's more at the appointment time when they're calling the patient.

Interviewer: So does the nurse call first _____, and then do you start the videoconference, or how does that –

Interviewee: Yeah, so the nurse will call first on the telephone, regardless if it's a telephone or a video visit. They will collect all of their information, and then they will let the patient know, your provider will be calling you with the technology, making sure the technology is set up. And then they end their call, and then the provider calls closer to the appointment time.

Interviewer: How did you implement those changes?

Interviewee: We had slowly, very slowly, started telephone visits. So we were – we were very novice in it still, but we at least knew the flow of telephone. For video, how we implemented it, we really just came together as an organization and said what technology platform are we going to use. We got all of our clinicians and care teams together virtually, and we did a WebEx of what is this technology, how is this technology going to work?

And then we also sat down as a primary care leadership team and said – with clinicians, and said, what is important for our nurses to be gathering? Is it the same as an in-person visit? Is there any aspects that you feel like we need to leave out or we need to add? So we made a grid that all clinicians and leadership agreed upon, and then we trained to that.

Interviewer: Which of the changes do you think have had the greatest influence on the quality of care for your patients with diabetes?

Interviewee: Can you say that again?

Interviewer: Yeah. Which of the changes that you've made have had the greatest influence on the quality of care for patients with diabetes?

Interviewee: I think taking into account patient reported blood pressures, since we have gone to this virtual platform, understanding that patient reported blood pressures can be taken into account when we are caring for our patients virtually. We had a lot of patients that didn't want to come into the clinic and were not going to come into the clinic, so we were still able to care for our diabetics, understanding what their blood pressure is at home, and comparing that to what we had in our EMR from before, and just continuing to care, even though we haven't taken the blood pressure. So that was a huge change, and it has allowed us to continue to focus on diabetes with all five measures.

Interviewer: Do you think that some of these changes are permanent or temporary? How do you see that happening?

Interviewee: I've wondered a lot of what the new normal will be. I think virtual visits are here to stay. I think we will continue to see our diabetics in a virtual setting, phone or video. I don't ever think that's going away. Our patients like it. Our providers are growing to like it as well.

The home blood pressures, I don't know. I don't know if that is temporary or permanent. I don't know on that one. But I think virtual visits are for sure here to stay with our diabetics, and they're loving it.

Interviewer: You included phone visits and video visits in your virtual visits. Are there differences?

Interviewee: From the quality of care, there should be no differences. From that relationship-building, I can say our clinicians feel – it's more of a connection when you can see that person, and they're also doing a lot of nonverbal assessment when they're seeing that patient. So yes, I think there are differences with the relationship piece.

Interviewer: You mentioned that some of your patients were resistant about coming in. Could you tell me a little bit more about how your patients with diabetes have been affected by COVID?

Interviewee: I would say speaking generally, when you look at our patient populations that have wanted to come in and not come in, our diabetic patients are higher risk, so they didn't want to come in. And I think they – hopefully, they haven't been affected, because we've been able to still offer them a platform to be able to connect with their patients. But they were very reluctant to come in, and our quality scores did actually drop with the A1C measure, because they didn't want to come in and get labs.

So we are recovering with the A1C measure, as we continue to show that this is safe. So I think if they were affected, it was continuing to keep up with their A1C, but we've done a lot of work around collecting blood sugars and making sure that we have as many blood sugar readings as we can. Video visits, you'll see patients hold up their notebook with all their blood pressure readings, so that's been nice, with the inability sometimes to get an A1C. So if they've been affected, it's been the A1C measure.

Interviewer: How did you reassure those patients, or did you address their concerns?

Interviewee: We did. We gave our providers – because that is the most powerful relationship, our clinicians and our patients, we gave – the clinicians knew what we were doing, but to give them the talking points so that when they are asked, the reassurance. And there was a lot of marketing efforts just in general to explain to our patients what we're doing in order to keep the clinic safe.

Interviewer: Do you think the patient – how do you think the patients have reacted to the video visits?

Interviewee: They like it. There's definitely patients that would prefer when they're booking their physicals as video visits. They found that the time factor is a lot easier, especially for our patients who may have constrictions around their schedule. They're not 40 minutes in, sit in the clinic, and then 40 minutes back to work.

So a lot of our patients are really enjoying it. There are still patients that want that face to face, and we are still offering that.

Interviewer: Do you think it's had an influence on your relationship, or has the relationship had an influence on the success of the visits?

Interviewee: I think we have found more and more that convenience can drive what the patient is looking for. So we are finding that sometimes the relationship, convenience may kind of trump the relationship

for the patient, and you see that with video visits. So I think it's been eye-opening for us, but the patients that have had a strong relationship with their clinician, they continue to maintain that. So it hasn't affected already established relationships.

Interviewer: What kind of – we'll turn a little bit and say what kind of help have you received from your health care organization to respond to the COVID pandemic, or that improves your care?

Interviewee: A lot of help has been given through the organization. I as the clinic manager didn't bring up video visits all by myself. That was an organizational technology. When we initially in March decided what are we going to do next, that was an organizational movement. How are we going to slowly bring back patients in? That was organizational. As an organization, we moved to respiratory and non-respiratory sites to help our patients that the clinician really felt like I need to see them in person, that we allowed that safe venue for the patients. So we did that as an organization.

The organization as a whole started a lot more of our batching of our outreach in masses to our patients around quality measures, and reminding them, you are due for a visit, you are due for your A1C. So the organization took a lot of that on, whereas it used to be care team specific. I mean, we have redone schedules as an organization, to ensuring that our patients have access from 7:00 to 7:00 when they need us, not just your traditional 7:00 to 5:00 hours.

So we've – as an organization, we have done a lot together.

Interviewer: Are there any things that you wish you had from your organization, or any other support that you'd like to get?

Interviewee: I don't think there's anything I'd like to get. I think I would like to take the batching back, because it's more of a personal touch. But I don't think there's anything I haven't gotten.

Interviewer: When we talked the first time, you talked about the importance of the proactive outreach and the care, and you've said that again now. Has that specifically altered? You've talked a little bit more about batching at the system level.

Interviewee: Yeah. I was wondering if you were going to ask me this question. I think COVID was kind of an experiment in itself that proved how much proactive outreach matters. When we almost felt like we

went into survival mode of how do we keep our patients safe, how do we continue to operate with a global pandemic, that outreach halted, and our quality measures showed that, significantly, XXXXX I feel like more than some other clinics.

So COVID did stop for an extended period of time that proactive outreach, and our measures have shown that. We actually just in the past month have started that proactive outreach again, and you see, if you're following just sheer data, those measures start to tick back up. So what we talked about a year ago really holds true, and we did a live experiment with it, whether we wanted to or not, and proactive outreach does a lot for our patients.

Interviewer: How did it – how did it stop, the proactive outreach? How did it affect it? What caused the problem?

Interviewee: What caused the stopping of proactive outreach? COVID. And just sheerly – us as an organization, we were I think at a juncture where we had to decide where do we focus our time and energy, and how do we keep our patients safe and our employees? And that really took 110 percent of our time to figure that out. And so the proactive outreach didn't get the time and attention it used to.

Interviewer: Sure. All the demands of the – all the educational demands, all the – I mean, staff? Did you lose staff? Anything like that?

Interviewee: Yeah, we – I personally furloughed two-thirds of my support team.

Interviewer: Well, then, okay.

Interviewee: Yep. So we lost staff. We were having – we had a lot of significant concerns about – well, we have team members that have some risk factors that they – coming into clinic was scary. We have clinicians with risk factors. Coming into clinic was scary. How do we do this and ensure that our patients are safe? We implemented video visits, that technology, and making sure all of our providers were up and running, and if our providers were working from home, because our care team spaces are not set up to social distance. So if a provider was working from home, how are they supported IT-wise? How are we going to set up the clinic so if I do have 19 providers in here, how are they socially distanced? How is my front line socially distanced? So there's just a lot of operational pieces that we needed to put into place in order to keep everyone safe.

You can think of the waiting room as well. We implemented self-

rooming, so our patients don't wait in the waiting room, and they don't feel like they may be sitting next to someone who's sick. So they just get right back there, and they feel safe. So all those were pieces we had to implement and shift our attention to.

Interviewer: Is anybody – when we started, you said you were kind of getting more back to normal.

Interviewee: Yeah.

Interviewer: Is that – has your staffing come back, or not, or is that –

Interviewee: Yeah, so our staffing is back to what we feel is normal, and like I said, April is a good perspective setting. It feels much better. I still – all 19 clinicians we started with, we still have all 19 clinicians. So clinician staffing has never been impacted. The amount of time they spend with virtual appointments versus in person appointments has shifted, and I feel like we're at kind of our sweet spot in our normal.

Interviewer: What is that? I mean –

Interviewee: So we're at –

Interviewer: Do you have an estimate?

Interviewee: Yeah, so we're at 30 percent for family medicine clinicians are virtual, and 20 percent – family med and internal med, and 20 percent pediatrics, for virtual, and then the other percentage is in person. And patients are filling that time and loving that time. We've leveraged technology to help remind patients of their appointments and help book them online seamlessly. So I feel like we are back in that normal to the point where we're focusing on that proactive outreach and bringing the patients in, whether it be in person or virtually, and letting the patient pick what works best for them.

Interviewer: That's fantastic. And congratulations for getting through all of this. It sounds like you're really responding to _____ a high functioning clinic. And is there anything else you'd like to tell me?

Interviewee: No, I can't think of it. Thank you so much for your time.

Interviewer: Well, thank you very much. And we'll make sure that we provide some kind of follow-up for you, too, so you can get an idea of where this is going. A bunch of articles have come out, and I think

some really wonderful information has come. We have a variety of really key stakeholders that are engaged. So thank you so much for helping us out.

Interviewee: Yes, absolutely. Happy Friday.

Interviewer: All right. Thanks so much.

Female: You too.

Interviewer: Bye.

Interviewee: Bye.

[End of Audio]

Interviewer: – if it's not...

Interviewee 1: I'm here. I am sitting in the visitor lounge at the XXXXX Hospital. My mom is – I think I told you guys that my mom had her hip replaced yesterday. So – I'm sorry, it was Tuesday. So, I'm still here so I will – yep.

[Background noise]

Interviewer: So, there's a lot – this may be challenging given the amount of noise in the background, but between the two of you we'll do our best. And I don't want to take a lot of your time, but again, thank you. This is the third interview we've done together. And we genuinely appreciate your time. We have a paper coming out based on the first interview and we're working on a paper based on the second interview. I had promised to share those with you.

So, I'm going to – the process is pretty much the same. I need to read a brief script and ask your permission to record and then we can get started. And we'll try to finish in 20 minutes or so.

Interviewee 2: Okay.

Interviewee 1: That sounds good.

Interviewer: Great. So, my name is Mickey Eder. I'm conducting an interview with the leaders of the YYYYY XXXXX and XXXXA and a few other clinics, I believe. It's October 29. The overall goal of the United study is to identify the specific changeable factors and strategies that are most effective in producing high scores on Minnesota Community Measurement measures for patients with diabetes. As you answer the questions please don't use patient or clinic staff names. I'd like your permission to record our conversation and then we'll transcribe it, and if there are any names used we'll remove them at that time. Do we have your permission to record this?

Interviewee 2:

Yes.

Interviewee 1:

Yes.

Interviewer:

Thank you. In our first two rounds of interviews our team learned that high-performing clinics like yours use proactive outreach to patients to address their areas of need as a main strategy. We're interested today in talking about how your diabetes care has been affected by the Covid pandemic. And I wonder if we could start by

having each of you briefly identify your main roles in the organization, both XXXXX and XXXXA, [name]?

Interviewee 2: My name is [name] and I am the patient care supervisor for XXXXX and XXXXA. In that role I manage the nursing staff as well as the medical assistant staff.

Interviewer: Thank you. X?

Interviewee 1: I'm finding the mute button. Here we go. Can you hear me?

Interviewer: Yes.

Interviewee 1: Hi. [ ] I am the clinic administrator for the XXXXX Clinic. I am responsible for the operations of the clinic, patient experience, quality, financial aspects of the site.

Interviewer: Well, thank you both. I wonder if we could start – and I want to ask how Covid has affected the way your practice – and if you can talk about – perhaps focus on XXXXA it might be easier since that was where we were last. And we were there because it had continuous improvement over three years. So, if you could talk about or at least distinguish which clinic you're talking about, it might be helpful. And we're interested in learning how Covid has affected the way your practice provides care for patients with diabetes.

Interviewee 1: All right. I think I can speak to that here. So, since March, as you can probably imagine, for the first – for March and April we hardly saw any patients at all. And currently what's happening is we are seeing patients that are due for physicals or that need to be seen. So, if a patient is diabetic but they're stable they're likely not coming into the clinic right now. We are doing phone visits and video visits with those patients so we can at least check in with them to see how they are doing. We do ask that they come in for a quick lab so we can get some lab tests on them. That's part of their visit as well. But they're basically in the clinic and out within ten minutes or so.

Only if they're not doing well or they have other concerns that we need to actually see them in person for – blood pressure problem, any symptoms that need an exam, we are scheduling them to be seen. But that is not our first option.

Interviewer:
Could you – so, the staff in your clinic, what is the – are people working from home or is everybody in the – what percentage are – what does the clinic look like on an average day?

Interviewee 1:
Yep. Yep. So, today is very different than it was a few months ago. So, we did go quite a few furloughs and providers working at home. Today we are all back to work. We are primarily seeing – basically everything that we are seeing in clinic is either a preventative type of appointment, a new injury, concerns if someone is sick that needs a – we need to listen to lungs or get an x-ray. So, we are all back at work today. And our goal is to do about 50 percent of our visits virtually, and we're at about – between 20 and 30 percent right now.

Interviewer:
And just – when you say "We're all back at work" is that approximately where you were FTE in February, let's say?

Interviewee 1:
So, at XXXXA and XXXXX one of the things that we are also going through is – now the overhead's on, sorry – is we're doing a little bit of a restructure. And so, we've had some positions vacate which we have not filled yet, but the intent is to fill them. The other thing I should probably throw in there about XXXXA is XXXXA has been identified as a site that will be closing. So, we will – our last operational day to see patients will be on December 4.

Interviewer: Well, there's lots of changes. Can you say what changes have you made in your care for patients with diabetes? Are there specific changes in the way you approach diabetes care given that you've described virtual and an emphasis on not seeing people in the clinic?

Oh, you're on mute. That's what –

Interviewee 1: Oh, sorry.

Interviewer: And it's hard to tell that you were talking because your mask is on.

Interviewee 1: Yes. I know. I wish I could take this off because it's hard to breathe. And [name] please chime in too if you'd like to add. But the process for taking care of a diabetic is exactly what I described earlier. We have primarily tried to keep patients out of the clinic and at home. And we're – the change is seeing them either video or some sort of virtual visit with a quick lab appointment if necessary.

Interviewer: Have you been – I'm sorry, I don't know if anyone else wants to add. Are you monitoring the same way? Do you know what the quality measures are looking like?

Interviewee 1: We do look at the quality measures on a monthly basis, and I can tell you for XXXXA – so, XXXXA was closed for – let's see, I think they closed for two, almost three months. Yeah. So, those patients would have been seen in either the XXXXX clinic or the XXXXB clinic if they needed to be seen or helped by a provider. So, that skews the reports a little bit when a patient is seen at a different facility.

So, what I can tell you in general, the XXXXA and XXXXX clinics have done fairly well – probably better than most – maintaining their quality measures. It has dipped a bit. But we still really are trying to focus on taking care of what they need when they're calling us. So, there is – I would say it's probably fair to say that the emphasis has – is still there but it's probably not as prevalent as it was prior to Covid. We really need to get back there and we're wanting to make sure that it's – we do it in a safe way.

So, we haven't – I guess to answer your question we have not initiated any new activity surrounding diabetics. XXXXX, am I speaking correctly on that? Or is there anything that…?

Interviewee 2: Yeah, I think that's correct. I think that we do also have a diabetic nurse educator who is doing some virtual visits with patients as well. I don't think she's bringing any into the clinic either. But that is another way that we take care of our diabetic patients.

Interviewer: How – can you say a little bit about the way the virtual phone and kind of balancing, having people minimally in the clinic has affected the way the care team coordinates and works?

Interviewee 2: The care team comes into the clinic. They do all of the virtual visits while in clinic and then they contact the patient over the phone. And then – it can either be a phone visit or a video visit. If it's a video visit they help the patient through signing up and getting – contacting them through the computer system. That is actually a better way because then the provider can see the patient. Although it's through a computer they can see what they're looking like and those kind of things. They do have the patients come in at times for our RN visits to have their blood pressures checked in between seeing them in the clinic. But all RN – or MAs contacting patients are in the clinic when they're contacting the patients. They're not doing that work from home.

Interviewer: Can you – and the providers as well? Or clinicians, I'm sorry.

Interviewee 2: The providers or the clinicians can do those virtual visits out of their home and some of them do. Some of that work is done at their home.

Interviewer: And are you finding similarities and differences – can you describe – you mentioned both video and phone visits, and can you talk about the kind of advantages or disadvantages of those interactions with patients?

Interviewee 2: I think telephone visits are sometimes easier for patients to maneuver through if they don't have the technology aspect in their home. But the video visits are a much better source for the providers to see the patient and work through and talk through some of their health maintenance items that may be do. But I think just being able to see them over the computer helps them to provide better care to that patient.

Interviewer: And can you talk a little bit about how your patients with diabetes have been affected by Covid and issues over the last six, seven months?

Interviewee 2: Well, just like [name] said, they're not coming into the clinic quite as often, so they are being seen virtually – so, either through the telephone or through the computer – and then coming in sporadically for maybe some blood pressure follow-up or lab follow-up. They do receive some letters sometimes if their health maintenance items are coming behind. If they are due and they are not up to date on those they do receive a letter which indicates to them what is due for them, to call and either make an appointment or make a video visit appointment to get those taken care of,

Interviewer: And –

Interviewee 1: I'm just trying to pull up our numbers too, [name]. And it's a little bit difficult when there's a live meeting going on, so I'll keep working on that. Are you asking in particular about any changes that we see trends with as far as Covid goes and the change in lifestyle that has occurred? Is that maybe what you're asking or…?

Interviewer: Yes.

Interviewee 1: Okay.

Interviewer: The latter.

Interviewee 1: Okay. I would not have that – I would have to do kind of some research in order to answer that question.

Interviewer: So, are patients as – so, you mentioned kind of helping patients connect remotely. Have you found that – are they interested in coming in? Are they interested in phone visits? Can you – we're just trying to understand the way in which there are preferences and maybe barriers to interacting and…

Interviewee 1: Yes. So, I think it's a fair thing to say, is it really depends on your demographic. And XXXXA, for instance, has quite a few, I would say, families. The average patient range – age at XXXXA is in the 40s, where the average patient age at XXXXX is more in the 50s, early 60s. Very different tolerance for virtual care. Those that are good with a smart phone, that have – that understand Zoom, maybe have some kids that use it seem to be okay with it. Others it's hard to navigate and it's not traditional, so they would – I think for the most part – and yes, we do have many that do video, but I would say that it's probably fair to say that the older generation prefers a phone call.

Interviewer: Phone than video? Or phone than in-person visit?

Interviewee 1: Yeah, we – like I said, before it – and being seen in person, we really are trying not to do that unless the patient just is flat out declining – just saying, "I'm not doing this." Then we will have them come in because it's more important to just see them than fight about the method in which we're going to see you.

Interviewer: Yeah. No, no. thank you. I'm just trying to be – make sure I'm following clearly.

Interviewee 1: Yeah.

Interviewer: Are there lifestyle changes that have occurred with patients that you and staff in the clinic are either acknowledging or aware of?

Interviewee 1: I think without doing a little bit of research to see which areas are – have fallen off a little bit it's my impression that the reason why these patients – our numbers being down are due to not having an office visit in the clinic because we didn't have a solution for that early on. So, patients need to be seen in order to meet some of the diabetic criteria, right? So, some of it is that. But I really

couldn't say anything else about the D5 criteria to see if there's any particular decrease in any specific area.

Interviewer: Two more questions. And I apologize to Rachel, who I'm not sure I acknowledged at the beginning. She may have a question also at the end. She has been involved from the beginning of this project. Going forward, would you – can you estimate what the relative proportion of in-person and video visits might be? Is there some goal that you want to pursue? Or are you just waiting to see how things play out?

Interviewee 1: I think as a whole from a primary care perspective for all patients we ideally want to get to a 50 percent virtual rate. And last I looked, we were between 20 and 30 percent right now and the majority of those patients are patients that don't need to come into the clinic that are fairly stable. So, that is just kind of passing along the directive, and the strategy for primary care is that we would like to be in that 50th percentile for our billable office visits to be virtual.

Interviewer: Would – is – were there additional comments anyone wanted to make? Okay. Last question that I have. And thank you; I can see the mask is a bit challenging and we appreciate you're willing to persevere through this. Each of the interviews we've concluded by asking about the help you've received from your healthcare organization, and in this case to respond to the Covid pandemic. And could you say something about how the organization has been – how you – what they've – what they have or haven't done for the – to advance your clinic, your patient health outcomes.

Interviewee 1: I can probably defer to [name]. I think one of the first things that I would mention is really about safety for ourselves and our patients. [Name] has been very much involved with ensuring that we are all practicing appropriate PPE practices. And I do have to say that our organization right from the get-go has had numerous – there's been a whole command center that has helped us through making sure we're doing things safely, consistently, that we are not in positions where we are making up things as we go. There's a ton of workflows that have been created both for frontline staff, providers, the whole Covid testing process, making sure that we are in the know about how to best get that done for a patient. Trying to stay – trying to create standard work versus having every clinic fending for themselves. So, [name], I don't know if you want to add anything there. I know you've been on meetings every week since Covid started in March regarding PPE and operational processes.

Interviewee 2: Sure. So, at each site we do have a PPE champion. They are ensuring that we have adequate supplies in each site, and that is, our supply chain is involved in this as well. We're doing some social distancing in our lobbies, making sure that we have screening at place at the front door. So, we have bouncers, we call them, our screeners at our front doors asking questions about associated symptoms. And if people are positive for any of those symptoms we make sure they have adequate masking on and then we do some rooming with the immediately so they're not sitting in our lobby. So, we have those measures in place.

The calls that she is talking about, they are involving infection prevention as well as our occupational health department is all involved in making sure that our staff as well as our patients are being kept safe. We have protocols in place for if our employees are calling in with symptoms. When you log into our Epic documenting system it tells you if you have any symptoms that you need to immediately contact the [acronym] department and then there's protocols in place for when they can return to work. So, kind of all of that has been progressively changing through this process and much work has gone on in standardizing that work across all of our clinics.

Interviewer: And without asking for real details, you've – it sounds like you've been testing for Covid and potentially had positives either in patient or staff?

Interviewee 2: Correct.

Interviewer: Okay. Rachel, anything else you can…?

Female: No, I think we covered everything.

Interviewer: So, without using names I'll just thank you both. I hope that you're not waiting too much longer and that things go well for the rest – with the recovery of people in your family.

Interviewee 1: Thank you so much for that. I appreciate it.

Interviewer: Thank you. And again, have a good day and be safe.

Interviewee 2: Thank you.

Interviewee 1: Okay.

Interviewee 2: You as well.

Female: Thank you.

Interviewee 2: Okay.

Interviewer: Bye bye.

Interviewee 2: Bye bye.

[End of Audio]

Interviewer: Right, right. Thank you. We'll get started since I know everybody’s always rushing these days. Does it seem to take longer to get things done where you are?

Interviewee: Yes and no, I was just talking with my supervisor, time is, the perspective is strange because if you look at the year it feels like it went by in a second, and it also feels like it took five years, instead of one. So it depends on how you’re thinking about it. It’s just a bizarre thing. We've squished so much stuff into a small amount of time. Things that normally would have taken us five years to complete we did sometimes in even just a couple of weeks. So it was, it was pretty phenomenal the amount of things that we accomplished this year. I feel like the leaped forward five years in technology even.

Interviewer: Well hopefully you’ll tell us a little bit about that response to the questions. Do you, you don’t remember Rachel, because she didn’t join me when I drove out to XXXXX. Rachel’s been with this project and I've worked with her before this project five or six years now. She’s been really integral to all this and, and may have questions as we go on. So I wanted to make sure, sometimes I forget to introduce her at the beginning and then I’m always embarrassed.

Interviewee: You’re not forgettable.

Interviewer: So, I’m conducting an interview today with RG at the XXXXX clinic, and last time we talked, you said that there was a clinic in the hospital as well that are kind of paired together.

Interviewee: Yeah, that’s, that’s actually where I’m at right now.

Interviewer: So, we’re conducting an interview the overall goal of the United study is to identify the specific changeable factors and strategies that are most effective in producing high scores on Minnesota community measurement measures for patients with diabetes, and I will apologize, I have to read the script, I should have said that right at the beginning. It’s an ethical requirement. And basically, we want to remind you, as you’re answering questions to abide by HIPAA rules about referring to specific patient or clinic staff names, if it’s okay with you, we’d like to be able to record the conversation will remove any identifiers if they get mentioned. Occasionally, staff refer to other staff by name and so on. So do we have your permission to record this?

Interviewee: Sure.

Interviewer: Thank you. In our first two rounds of interviews, we learned that high performing clinics, this is part of the answer to your question, like yours,

use proactive outreach to patients to address their areas of need as a main strategy. Today we're interested in talking about how diabetes care has been affected over the last year by the COVID pandemic. If we could start by having you identify your main role at the clinic that would be great.

Interviewee: Yep, I'm the clinic manager.

Interviewer: What does that mean?

Interviewee: it's kind of the oversight I guess of the whole picture. So, the staff on the clinical side will report up through a clinical supervisor, the front desk and business staff will report up through the business supervisor, and then they report up to me.

Interviewer: You have responsibilities for both clinical and operations.

Interviewee: Yep.

Interviewer: Thanks. And could you say, how COVID has affected the way your practice, and so let's just talk about the clinic if that's okay, the way that the clinic provides care for patients with diabetes.

Interviewee: Yeah, so I think we had kind of started to tip toe into it a couple of years ago just barely because we are an innovative clinic. We were kind of looking for more technology. When COVID hit, we had to dive in headfirst into virtual care. What ended up happening here in our situation is, because we have one clinic that is part of the hospital and one that is not, we had to create space. In case there was a significant surgery we just didn't know where things were going. As well as needing to preserve PPE, as well as giving people a break. And so, with those three things in mind what ended up happening is we closed down the primary care clinic that is on the hospital campus. And we joined them with the other clinic, in addition to another nearby YYYYY clinic. We had three clinics worth of providers and staff within the two physical buildings. They were combined together. And then rotated every other week between my site that became the respiratory clinic, or anybody that had respiration symptoms, which was likely going to be covid, and then the other one was what we would consider like the well clinic, people that are coming in for diabetic care, you know, things like that. So that's where it kind of initially started and literally rotated every week, the providers and staff moved back and forth between the two.

And so, you want to talk about burnout, exhaustion, workflow changes. It was pretty crazy and when we did that, we also went headfirst like I said, into the virtual care because, as much as possible you want to keep those covid patients at home. And so we got all of our providers immediately set up to be able to do virtual visits. And that way, you know if they were a

patient that felt that they were medically fragile, then they could do a virtual visit, or if they were a covid patient and were too sick, and/or just to keep other people not exposed, we can keep them at home and see them virtually as well. So the challenge became as you are rotating between focus being on respiratory, the next week now you're focusing on chronic care. Right? All the diabetic, all those different ones to keep shifting your brain, back and forth every week, working with people you don't, you've never met before you don't even know because they combined with other clinics. We had combined leadership, things like that. It was very, very difficult during that time. Very, very difficult.

Interviewer: When you say during that time how long did that process, can you give us an idea of the timeline?

Interviewee: That's a good question. That went on for probably, I'm going to guess. This is me taking a stab at it, off the top of my head, maybe three months. And then we were able to reopen our clinic, and we stopped the rotation. We've gotten enough PPE in place. You know we got plexiglass up on our counters. We got the protections for the patients, we've got the signage up, we've got the six fee spacing you know indicators, things like that. So once we got everything in place that we felt that we were safe and we could keep our patients safe were able to reopen our doors in what would be a more normal sense of able to see, you know, diabetic patients like we did before. So you know the COVID patients, you guys are sitting over here and those that have no symptoms, you're sitting over here so there is, you know, a semblance of safety. And then, of course, anybody you know that we needed to, we would get them back as quickly as possible into exam rooms so that there wouldn't be people waiting in the waiting room.

Interviewer: So sometime in the middle of the summer you'd say you went back to not way things were before but providing a full range of services in one location. Is that fair?

Interviewee: Yeah, I would say it's right about the same time that New York's numbers started coming back down, because during that we also had one of our clinical supervisors was deployed to New York. So during all that we went down a leader. So she was gone, and just as we stopped that rotation that's right when she came back. So it kind of worked out perfectly as we got two buildings back again.

Interviewer: So just to be a little clearer, did both clinicians and staff rotate? Did they stay as kind of units and teams?

Interviewee: Yeah, so who they normally work with as a rooming staff person, we would try and keep them with them so there was as much possible as we could. Because it almost creates a safety issue, right? When you're now

being thrown into a clinic that you've never worked in before. You know where's the supplies, and where do you go when you have an issue, and where do you hang your coat, even. Right. So, as much as we could we tried to keep continuity, as much as we could.

Interviewer: Thank you for clarifying. Speaking specifically about the care of patients with diabetes. Can you describe whether there were changes and what kind of changes may have occurred? And again, things may change over time but however you think is the most appropriate we're just trying to understand how the...

Interviewee: Yeah, so the care itself, so we we've been doing this for a very long time. So, you know, calling patients, you know, trying to hold them accountable, utilizing diabetic ed, you know, all these different resources that we have. We would still do our absolute best to do that. We ran into barriers. The barriers are if we felt they needed to come into the lab they may not feel safe to come into the lab. They don't want to get exposed to covid. Secondly, is some didn't find value in virtual visits. It is not the same as being physically listened to your heart and your lungs you know by the by the provider feel that, like you're not getting your dollars worth, right?

Additionally, every time that a staff member, or provider was potentially exposed, we were ending up in the having staff or providers going into quarantine all the time. And so we had staffing shortages, as well as all this upheaval of rotation and then coming back and still doing a significant portion of virtual visits. Still are doing a lot, and that, like I said, I think that we've jumped five years in technology. We were kind of going in that direction anyway, that's kind of where this generation is. But we kind of forced it on the older population which has some of these comorbidities, right? We kind of forced it on them and said you have to do virtual visits, we gotta keep you safe. And so it was it was it was a forced jump in technology in a very short period of time. All these things that we were doing like we closed the clinic and we started this rotation is totally do that it was like, you know…[lost audio for a few seconds] There we are.

Interviewer: So, you were saying, how… were you doing many virtual visits before, let's say March?

Interviewee: Prior to that that we had this a couple providers that were interested in it. Like I said we are a very innovative clinic. I'm kind of a technology geek, to some degree, in so anybody that was interested in it at all, I was getting them cleaned up so the perfect part is that I had already been initiating getting more people up on virtual visits when this happened. So we have our clinics, compared to others and we have a little bit of a leg up because

I was already in the process of getting the providers trained and getting the, you know, we have to get the webcam and, you know, the microphones and all things without for all that stuff. We were already getting all that in place. And so, we're a little bit lucky in that regard for the size that we are, it would have taken a lot, lot more work.

Interviewer: Could you remind us how many people you're talking about, clinicians?

Interviewee: Between the two clinics I think I've got eighteen of just primary care providers but then we've got mental health providers too. With this primary care providers between the two there's about 17, 18.

Interviewer: When the clinicians had to quarantine did they see patients at home virtually or…

Interviewee: Sometimes, if it was just an exposure and they had no symptoms then that was something that we did consider or look. You know we didn't force it by any means you know, maybe they're taking care of a loved one at home, we're not going to, you know, say you have to virtual visits, you know, it was up to them. In most cases they wanted to, they were feeling fine they did it, you know, I've got one provider, I mean, they're getting stressed out they are exhausted, their kids are having to stay home from school you know all these different things that are going on it's not just happening to, you know, the staff it's happening to the providers, they were, there's no way to stay home with their seven and eight year old that now got sent home from school and you gotta do some hybrid version or virtual. They're trying to figure it out and so they themselves are getting a little burned out and frankly we're trying to figure this all out. And so, some of them elected to remain with having maybe one day a week where they stay home and do it virtually from home. You know, some do, you know, hours scattered throughout the week, things like that. Sometimes have it mixed throughout their day. You know, a lot of times they'll watch their, their schedule and they'll say okay this one has got you know respiratory symptoms let's give them a call and see if we can get them to do virtual visits instead of coming in. So we actually do pretty high volume or virtual visits yet.

Interviewer: Were you able to continue running your monthly quality reports?

Interviewee: Yeah. Thankfully we do have, our clinical supervisor is very dedicated to that. Despite that, because of the lower numbers of staff, we ended up having a little bit harder time doing some of those babysitting things that you do, you know, calling them up and saying, "Hey, you're due for your lab, hey you're due for your appointment. Hey, you know, you're labs are off and please do this…" So, a little bit harder to get people to comply and a little bit harder to actually do some of that work that you do every month

to, you know, remind them again. Because of that, number, I feel did fall off. We're usually very high in our scores. And this year, it looks a little more red and green at the end of the year we're normally, pretty much all green.

Interviewer: So, that's the answer to the question you asked before we started the formal interview when I mentioned proactive care. That was an approximation of what you termed babysitting, or reaching out or just reminding people that they were due things that they needed to tend to things. Have people been able to see the educators, and so, on similarly?

Interviewee: You know, I haven't followed up with them to see the how well that's going, but my understanding is that it is going well. We did have another situation that we're kind of working on with our diabetic. Where in we don't have endocrinology up here, and the XXXXB clinic is the closest that we would refer to. And of course they're having a really hard time fitting everybody in there's just not enough endocrinologists and so just recently we are embarking on a pilot with XXXXB some of the other clinics in the area, where if we refer a patient to endocrinology that's diabetic, what they'll do is sometimes it takes a while for them to get in but of course we want to get them started right away on things and so what we're doing is, in the meantime while they're waiting for the endocrinology the diabetic educator and them are reaching out getting them going on things, meeting with them as necessary to kind of give them a leg up, to get them going before they meet their endocrinologist. That is just too much time for them to be waiting to get into the endocrinologist. And that just started, probably in the last, gosh time is still messed up, I'm going to take two, three weeks, something like that.

Interviewer: And you had emphasized, when we spoke before, you had used the term Quick Starts where you wanted to newly diagnose the people who met with the educators almost immediately.

Interviewer: And that is actually, the Quick Starts are actually our RNs that are in our clinic. So the RNs in our clinic typically focus on accountable health and things like that, but if we have a diabetic patient that we need to a Quick Start on, we reach out to the RN, and they can get that going. And then they follow up with a diabetic educator.

Interviewer: So are there any things other than, you've talked some about the demands and the challenges, are there changes in the clinic you think are going to be permanent that will influence the care for diabetes maybe we just mentioned the endocrinologist situation.

Interviewee: Yep, the endocrinology situation. I think that the change is very good at best just our specific region that's trialing that, but I can see where that's

probably going to get rolled out to photograph, as well as the virtual care, some people…We had a doctor this morning who unfortunately had to tell the patient that I'm sorry we cannot do this all right now. Patients are really busy, so busy. This particular patient decided to call while they were driving doing video visits, but it is like no no no, we can't do that. But that's where we are at at, you know, and the guy was actually upset because he's like "this all the time I have, I don't have enough, I gotta drop of my daughter at school, I go to work and I got it all the time I guys, don't you know" And the doctor is still like "I'm sorry we cannot do that that's distracted driving, that's not appropriate." So it did end that visit, but that's where we're going with a lot of this as our generations change into being more computer savvy, you know, with our smartphones and things like that. Life is getting a little more casual as well. People are expecting things quicker. We had, we've had a little situation where some patients are a little too casual, where maybe they're not feeling well or whatever so they're still laying in bed with their jammies on! But, yeah, yeah. So we will have to get that fixed.

So the one thing that's a little frustrating. There's a transition piece that's happening. And it's really kind of a bummer. There are patients that sometimes they just need to talk through where whatever is going on, you know to say "hey my blood sugar been running a little high" or whatever, you know, and the doctor can make that change based on the conversation. They don't necessarily need to physically see them they just need to talk with them through whatever going on and then they can make an order. Currently, we are able to get some reimbursement from phone visit in my understanding is that in future that reimbursement is either going to be decreased significantly or gone. And so that's a major bummer for these people that can't quite get that video thing figured out but they can certainly get a phone call figured out. And that's going to be huge loss I think, because that actually is something that the patients really liked and when they can't get that video things figured out, if, you know, we were looking at a phone call and then they could still get them here taking care of. Once that's not reimbursed any longer, that's not going to be able to easily switch to and say, "Oh, don't worry about it you know I just need to talk to you about it here let's try this instead." That's not going to be a thing. And so I think that's a loss for us to some degree because especially in the older generation, which are your diabetics a lot of times, they're just not as savvy with the smartphones and video and things. So, the phone call works really well for them and so I think it's a bummer that we're gonna, that we're gonna lose that.

Interviewer: Have you been doing more lab only visit so you can talk to people virtually on the phone and then we need.

Interviewee: Absolutely. So what they'll do is they'll do. So we're trying to keep them out of the clinic as much as possible. Just to have the lowest amount of exposure possible, right? So they'll do a virtual visit, you know, with a provider and then they'll just come in for the lab. Yeah, absolutely.

Interviewer: So how have the patients themselves been affected?

Interviewee: You know, I haven't talked with any of them, other than they end up coming to me when they're upset about things, which, the good news is that's not something that they've been coming to me with, so I'm feeling like they must be okay with the process and, you know, reading through patient comments and things like that and the way things are being handled has not been an issue so I think they're appreciating the ability to do virtual visits, whether it's video or phone, and just coming in to get the quick labs done things like that. We also have increased our ability to do scheduling through my chart and things like that too. And we're now releasing lab results and things like that pretty much immediately from…uh… if they have their MyChart accounts and things like that they …

Interviewer: They don't go to the doctor and the doctor reviews them and releases them, they go immediately into the…

Interviewee: It's a new law that things get released immediately. There's certain ones, obviously, that are very touchy, not released immediately. But all the ones that that were identified they now have to be released immediately. And so now we're having to do a little bit of expectation setting and say, "Hey, you're going to see results. Give us a little bit of time, the doctor will look at them and will respond." But yeah they're getting the results before the doctor can even respond very frequently at this point, they're released immediately.

Interviewer: So, a little more broadly, you just were describing the patient's experience of receiving care. How do you think, if you think about the rest of your life, you described a little bit about shortage of time and having to juggle lots of things, as we all are. Do you think there have been particular health related aspects, whether it's behavior...

Interviewee: Yeah, This is a side topic I guess. They all go hand in hand. The thing that I'm seeing more out of this whole situation is that mental health has been greatly impacted. The amount of patients that I have that are, unfortunately, I don't know what else to say other than calling it like, kind of going off the edge. Like you know they were kind of right on the edge before they've been weary, they've been dealing with all these different things, you know, socially isolated, the fears of covid, the politics, now the darkness of winter, holiday stress that normally comes on. Some of these

patients, I can't even tell you how horrible…they are going off the edge, it's the kindest way I can say it. We're having to get police involved, I'll just, I'll say it that way. And so of course you know if they have diabetes and things like that on top of it. That's, that's going to be greatly impacted if they've got diabetes, and now you've got mental health issues, you're not going to be getting the care that you need. I can't even tell you, I've never seen it like this. I've done leadership for a long time, and the mental health aspect of this is just phenomenal. It's very, very sad.

Interviewer: And is that something you provide, you have a mental health component?

Interviewee: Yeah, we do. Yeah, we do have, we do have psychology here, but they're not physically onsite anymore. They are doing that 100% virtual at this point. I know that some of them are starting to come back into the clinic, but, up here anyway, they are 100% virtual. One thing that I don't know if it'll happen but we're trying to reach out to see if we can get a mental health coordinator. So, when we have some of these patients that maybe can't get into psychology or psychiatry right away that they can at least reach out to the patient and start having some conversations and, you know, doing some initial therapy. So, that isn't completed yet. We don't have that person physically here and I don't even know for sure if it got approved. But we definitely put it out there and hope to get that. So, definitely, definitely need I wish we had, you know, they asked us if we want it I said yeah six months ago.

Interviewer: Well thank you for sharing that, I know it's been a challenging time. One last question related to the role of YYYYY in helping you respond to the pandemic, or perhaps not. How has the larger organization contributed to your activities?

Interviewee: I tell you what, as chaotic as it has been, I'm really glad to be with YYYYY. I can't imagine being in a smaller organization and trying to accomplish the things that we've accomplished this year. It's been pretty phenomenal. I have been outside of YYYYY in the past and I know what it's like to not have that support in place. To have a legal department to have an infectious department and a risk and safety, those committees, those people are sometimes driving us crazy, but they are keeping us as informed and as safe as they can. So I'm not out here trying to reinvent any wheels, they get to reinvent the wheel.

Interviewer: How do they communicate with you?

Interviewee: Yeah, but it comes in various ways. When we were hot and heavy into that rotation and things were brand new, we were getting multiple ways of communication, sometimes it's just flat out email that says here's your covid update. We have an intranet, which is our own YYYYY focused

area on the internet, and only employees can see. That that has all of our private information, you know what is the current PPE to be wearing what are the current workflows, because everything has to be constantly changing. We would have these phone calls to say 'hey watch for this." And, you know, it would be coming down through, what we call them huddles. We have huddles every day at various levels, so I go through multiple huddles everyday to make sure that the communication is going across the board. I learned something probably at every huddle. They're definitely valuable. YYYYY itself has tried to, it was going to in 2020, do what is called HRO "High reliability organization". And so we are trying to roll it out and it's been a little bit challenging, because you obviously can't meet classrooms at this point. But a lot of us have already gotten trained, and we're continuing to train and instead of having a deadline of the end of this month, which is going to be the end of the year we've expanded that out in the spring and summer so that we can all do everything as safely absolutely possible. That's one of the things that we found is when you are low staffed, you're so busy, your workflows are changing every single day, sometimes hourly, it's really easy to not stop and do your double check. You know, and so having this again training and the real emphasis on safety of being a high reliable organizations and say, 23, two three, so there's no misunderstanding. I got one sitting right here, a little thing that tells us the military letters you know G is golf. And so as you're talking to somebody, they're not going to misunderstand what you're saying, from a number of perspective from a letter perspective, doing those double check all those different things. So YYYYY has been really great about doing their best to keep us safe. And in a very difficult time to do that.

Interviewer: One last question, and I appreciate you spending so much time. You mentioned being short staffed. Were there particular staffing changes that were either locally or organizationally determined? Or are you just....

Interviewee: Yeah, all of the above. So one is just flat out quarantining. So when the staff had been exposed, or came up positive with symptoms, things like that, then they would have to quarantine. So that in itself caused a staffing shortage. To keep us fiscally responsible we also decreased our staffing ratios, as well, to just ensure that that wasn't going to be a problem. We want YYYYY to still exist in the end, because our volumes dropped crazy low in March and April. People were scared to death to come in, you know streets were empty if you remember. So we did drop our staffing ratio for a bit. Still a little bit low. So the other problem that happened, and I just read an article about it and it's true for us too, is that people that have the option to not work, physically in a building are taking that option. So, for example, we had some business staff, we had an opportunity,

which was already going in place where people can go and work from home. Well, when this hit of course they all signed up for it. They're like “Well yeah my kids are at home. This is the best option for me, plus it gets me out of the risk of getting covid,” so they would take those options. Then when the openings came up, those openings are hard to fill, because of same thing. You know mom or dad or whoever is staying home with the kids and, or, you know, just staying out of the way of covid because they don’t need the money. They're opting to not take the open position> you know, and I just read that in an article, that’s happening all over there’s all kinds of open positions everywhere.

Interviewer: So you had mentioned previously the XXXXX is far enough away from an urban center that it makes it a little challenging to attract people. And I'm assuming that hasn’t changed?

Interviewee: Nope, we are still in the same location. Sorry couldn’t help that one. No, we have, we did do some incentives, you know, hiring incentives and things like that to see if we can help in this YYYYY-wide. It’s not just XXXXX but everybody is having issues with hiring right now. So yeah from an YYYYY perspective they added something hiring incentives, and we’re doing everything that we can. We’ve reduce some of the things that we've done in the past during our hiring process to just reduce the time to beat out our competitors, things like that. So yeah we’re doing our best. We did just hire somebody just recently we’re pretty excited about

Interviewer: Well is there anything that I haven’t asked that you want to talk about as far as addressing challenges over the last year?

Interviewee: Furloughs were challenging. I forgot to add that part. There was a lot of furloughs that occurred. And that was part of our staffing challenge as well. So, and then to add to that, the furloughs when you know people were gone for a month, two months, that would be equivalent to being gone for a year because everything has changed. everything had changed. So it was like, I had several staff say to me it was like starting a brand new job, because everything is changed so dramatically from being out on Furlough.

Interviewer: So you are close to where you were before the pandemic now in terms of staff?

Interviewee: Yep, all the staff are back. We don’t have anybody on furlough or anything like that. But we do have some open positions that are challenging to fill. Yeah, we’re getting there,
